# Phase‐Pure 1T’ Molybdenum Disulfide Synthesis and Stabilization

**DOI:** 10.1002/smsc.202500107

**Published:** 2025-03-21

**Authors:** Zongliang Guo, Hao Cheng, Ming Yang, Chi Ho Wong, Tawsif Ibne Alam, Shu Ping Lau, Yuen Hong Tsang

**Affiliations:** ^1^ Department of Applied Physics The Hong Kong Polytechnic University 11 Yuk Choi Rd, Hung Hom Kowloon Hong Kong SAR 999077 China; ^2^ Shenzhen Research Institute The Hong Kong Polytechnic University Shenzhen Guangdong 518057 China; ^3^ Photonic Research Institute The Hong Kong Polytechnic University 11 Yuk Choi Rd, Hung Hom Kowloon Hong Kong SAR 999077 China; ^4^ Research Institute for Advanced Manufacturing The Hong Kong Polytechnic University 11 Yuk Choi Rd, Hung Hom Kowloon Hong Kong SAR 999077 China; ^5^ Division of Science, Engineering, and Health Studies School of Professional Education and Executive Development The Hong Kong Polytechnic University 11 Yuk Choi Rd, Hung Hom Kowloon Hong Kong SAR 999077 China

**Keywords:** 1T’ molybdenum disulfide, hydrogen evolution, self‐intercalation, stabilizations, transition metal dichalcogenides

## Abstract

Metastable‐phase transition metal dichalcogenides (TMDs) show distinct structures and properties compared with the well‐studied thermodynamically stable phase. The phase impurity and degeneration are two critical challenges for the research and applications of metastable 1T’‐phase MoS_2_. Here, a self‐intercalation method is demonstrated to synthesize and stabilize the phase‐pure 1T’ MoS_2_. The K_2_S intercalation and 1T’ MoS_2_ synthesis are simultaneously done in only one step, leading to uniform intercalation and 1T’ phase purity. This engineered intercalation structure achieves stabilization of 1T’ MoS_2_ without changing its in‐plane structure. It keeps 1T’ phase structure and 100% phase purity even after 750 °C annealing or 1‐year aging exposed to air, while 1T’ MoS_2_ transforms to 2H phase gradually, or instantly over 97 °C. The theory calculation results show that the K_2_S intercalation lowers the formation energy and makes metastable 1T’ phase become stable. As a result, this stabilization method prevents gradual degeneration of applications performance that is inevitable in the past. This mass‐production‐available method has been successfully proved versatile for various 1T’ TMDs with numerous alkali metal chalcogenides intercalation. It eliminates a significant disadvantage of 1T’ TMDs, which can facilitate the investigation of novel properties and the development of fresh applications.

## Graphical abstract

The synthesis and stabilization of phase‐pure 1T’ MoS_2_ or other transition metal dichalcogenides (TMDs) are achieved by a self‐intercalation method. During this mass‐production‐available process, intercalation and formation of 1T’ TMDs are conducted simultaneously, leading to uniform defect‐free intercalation without changing in‐plane structure, and phase purity. The intercalation lowers the formation energy and makes metastable 1T’ TMDs become stable.

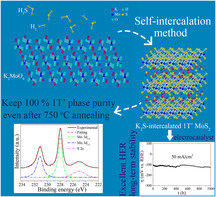

## 1 Introduction

Transition metal dichalcogenides (TMDs) have drawn tremendous attention recently due to their special layered structures and unique physicochemical properties, like graphene. TMDs family is not only a group of compounds; each compound usually has several phases showing different properties.^[^ ^1^ ^]^ The most well‐known and studied phases are thermodynamically stable, such as 2H phase MoS_2_. As other phases have higher system energy, they are usually unstable and easily transform to the stable phase, making the synthesis of metastable phase TMDs with high phase purity and crystal quality a huge challenge.^[^ ^2^ ^]^ Among several metastable phases, 1T’ phases have attracted the most attention. Compared with the stable‐phase TMDs, the metastable phases possess distinct electronic, optical, and electrochemical properties and consequently promising application potential in superconductivity,^[^ ^3^ ^]^ devices contact,^[^ ^4^ ^]^ surface‐enhanced Raman scattering,^[^ ^5^ ^]^ energy storage,^[^ ^6^ ^]^ electrochemical catalysts,^[^ ^7^ ^]^ supercapacitor,^[^ ^8^ ^]^ and synaptic transistors.^[^ ^9^ ^]^ To explore and achieve their potential applications, preparing these metastable‐phase TMDs with high phase purity and stabilization is necessary. The phase transition of group VIB TMDs from stable 2H phase to 1T or 1T’ phase by chemical treatment,^[^ ^10^ ^]^ electrostatic gating,^[^ ^11^ ^]^ and applying mechanical strain^[^ ^12^ ^]^ was reported, but its low phase purity (up to about 80%) causes poor performance, and these methods require strict conditions which makes them not suitable for mass production. Except for the phase transition method, direct synthesis of metastable phase TMDs by colloidal reaction^[^ ^13^ ^]^ and solvothermal method^[^ ^14^ ^]^ has also been investigated. However, the low phase purity and crystal quality are critical drawbacks. Recently, chemical oxidation of alkali atom intercalated TMDs which have a similar structure to 2H phase,^[^ ^15^ ^]^ was utilized to obtain 1T or 1T’ phase group VIB TMDs. The alkali atom intercalated TMDs are mainly formed by annealing of A_2_MS_4_ (A = Na, K, M = Mo, W) in H_2_^[^ ^16^ ^]^ at 850 °C or by the reaction of A_2_S, MS_2_, and M (A = Li, Na, K, M = Mo, Nb, Ta, Ti)^[^ ^17^ ^]^ at 800 °C. This method can achieve around 90% phase purity, but the postchemical treatment usually causes crystal defects. Moreover, the 1T or 1T’ phase TMDs have an inevitable short lifetime even at room temperature, as they tend to transform to stable 2H phase gradually,^[^ ^18^ ^]^ and it is accompanied by gradual performance degeneration. The stabilization of metastable‐phase TMDs without changing their intrinsic properties is a tricky problem. Introducing alkali atom intercalation is an alternative method to obtain thermodynamically stable 1T’ phase TMDs, but the chemically active alkali atoms cannot remain stable in air and cause transition metal oxidation.^[^ ^19^ ^]^ And the high 1T’ phase purity requires a high alkali atom intercalation ratio which leads to reduction of TMDs and the formation of transition metal.^30^ The syntheses of 1T’ TMDs with high phase purity and stabilization are still great challenges.

Here, a self‐intercalation method has been developed to prepare and stabilize phase‐pure 1T’ MoS_2_ with K_2_S intercalation. It is thermodynamically stable and inerts in air, ethanol, and even water. The 1T’‐phase MoS_2_ with high crystal quality was confirmed by Raman spectra and transmission electron microscope (TEM) results. The X‐ray photoelectron spectroscopy (XPS) characterization showed 100% phase purity. Thermogravimetric analysis (TGA), differential scanning calorimetry (DSC), and the annealing test results indicated thermal stability over 750 °C, while intrinsic 1T’ MoS_2_ transforms to 2H phase at 97 °C.^[16a]^ The synthesized sample has remained in the 1T’ phase and kept phase purity even after a 1‐year‐long aging test, exposed to air at room temperature. To evaluate the stabilization effect on applications performance, the K_2_S‐intercalated 1T’ MoS_2_ nanoflakes were synthesized by this method and used as an electrochemical catalyst in hydrogen evolution reaction (HER). It showed a comparable HER performance compared with the best reported TMDs‐based HER electrocatalysts and nonprecious HER electrocatalysts, which indicates that this engineered intercalation structure does not change 1T’ phase properties. Importantly, the 1000‐h and 30 000‐cycle HER stability provides an overwhelming advantage among other best TMDs‐based HER electrochemical catalysts, usually metastable 1T or 1T’ phase. The Na_2_S and Li_2_S intercalated 1T’ MoS_2_ were successfully synthesized by the same method. K_2_Se‐intercalated 1T’ MoSe_2_, Na_2_Se‐intercalated 1T’ MoSe_2_, and K_2_Te‐intercalated 1T’ MoTe_2_ were also obtained, suggesting that this method can be used to synthesize various 1T’ phase TMDs.

## 2 Results and Discussion

### 2.1 Self‐Intercalation and Synthesis

**Figure 1:**
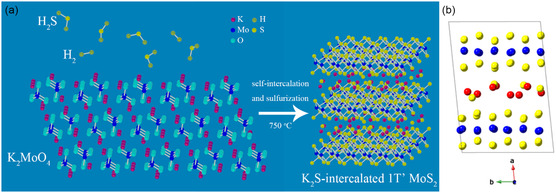
a) Schematic of self‐intercalation and synthesis. b) Atomic model of K_2_S‐intercalated 1T’ MoS_2_.

**Figure** **1** shows the schematic of self‐intercalation and synthesis of 1T’ MoS_2_. The dipotassium molybdate is heated to 750 °C, and then the mixture gas of H_2_S and H_2_ flow into furnace chamber to sulfurize the dipotassium molybdate. The intercalation of K_2_S and formation of 1T’ MoS_2_ can be simultaneously achieved during the sulfurization at 750 °C. After 4 h reaction and subsequent cooling to room temperature, the K_2_S‐intercalated 1T’ MoS_2_ is obtained without any post treatments. To synthesize Na_2_S‐intercalated 1T’ MoS_2_ nanosheets, the K_2_MoO_4_ powder is simply replaced with Na_2_MoO_4_ film on sapphire substrate prepared by sputtering deposition. The full details of the synthesis process are given in the Experimental Section. The sulfur supply is carefully controlled to make the reaction only happen at 750 °C, avoiding any low‐temperature reaction. It is found that self‐intercalation of K_2_S only happens at around 750 °C, and low‐temperature reactions lead to phase impurity and 2H phase formation. As the attempt to prepare K_2_S‐intercalated 1T’ MoS_2_ at lower temperature failed, it is believed that the K_2_S requires energy to locate themselves between 1T’ MoS_2_ layers. The 1T’ MoS_2_ belongs to monoclinic crystal system and C2/m space group. Interestingly, dipotassium molybdate (K_2_MoO_4_) also belongs to the same crystal system and space group. Figure 1a shows the structure of dipotassium molybdate, and the schematic of self‐intercalation and synthesis, the atomic model of K_2_S‐intercalated 1T’ MoS_2_ is given in Figure 1b. There are layer‐like distributions of molybdenum atoms and potassium atoms in dipotassium molybdate, corresponding to layered 1T’ MoS_2_. This structure similarity would be one reason for self‐intercalation. Considering the fact that the only source of intercalated potassium element is dipotassium molybdate itself, this self‐intercalation method can achieve uniform intercalation stoichiometrically and spatially. During the synthesis, there is no solution‐phase intercalation. The products are directly grown without any post treatments, so the typical defects caused by solution‐phase intercalation methods do not appear, thus leading to phase purity and high‐crystal quality. It is worthy to note that density functional theory (DFT) calculation results indicate K_2_S intercalation lowers the formation energy of 1T’ MoS_2_, which makes metastable 1T’ phase become thermodynamically stable and allows 1T’ MoS_2_ synthesis at high temperature. The following discusses the characterization and stability of intercalated 1T’ MoS_2_ and gives evidence.

### 2.2 Characterization of Intercalated 1T’ MoS_2_

**Figure 2:**
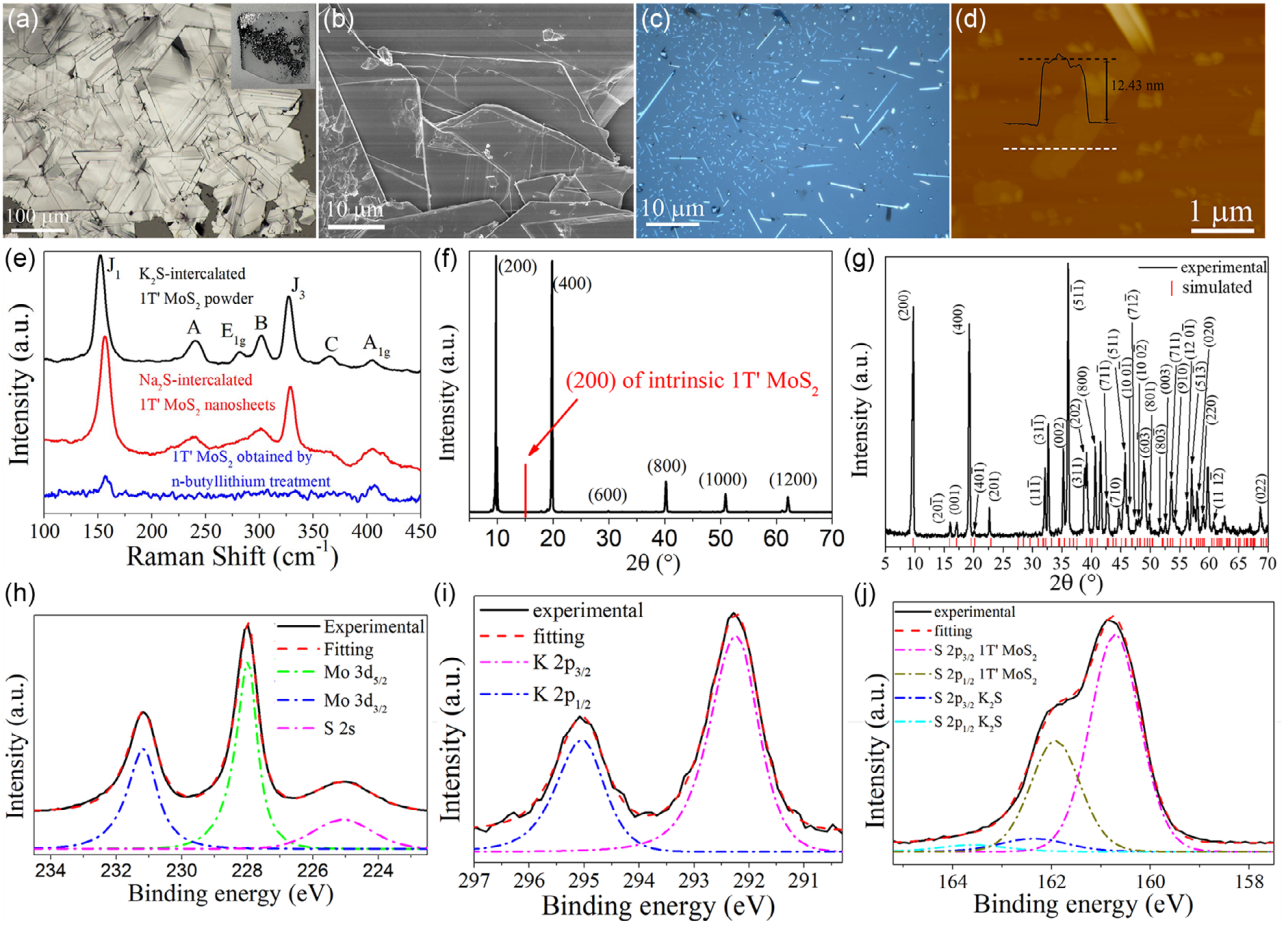
Characterization of K_2_S‐intercalated 1T’ MoS_2_ powder and Na_2_S‐intercalated 1T’ MoS_2_ nanosheets. a,b) Optical and SEM images of K_2_S‐intercalated 1T’ MoS_2_ powder, respectively. Inset of (a), photo of K_2_S‐intercalated 1T’ MoS_2_ powder on the sapphire substrate. c) Optical image of Na_2_S‐intercalated 1T’ MoS_2_ nanosheets. d) AFM image of Na_2_S‐intercalated 1T’ MoS_2_ nanosheets and height profile along white dot line. e) Raman spectra of K_2_S‐intercalated 1T’ MoS_2_ powder, Na_2_S‐intercalated 1T’ MoS_2_ nanosheets, and 1T’ MoS_2_ obtained by n‐butyllithium treatment. f,g) XRD results of as‐synthesized and fully stirred K_2_S‐intercalated 1T’ MoS_2_ powder, respectively. h–j) Experimental and fitted XPS Mo 3*d* and S 2*s*, K 2*p*, and S 2*p* spectra of K_2_S‐intercalated 1T’ MoS_2_ powder, respectively.

As shown in **Figure** **2**a,b, the as‐synthesized K_2_S‐intercalated 1T’ MoS_2_ powder consists of many trapezoid flakes stacking together with single flake maximum size of over 100 μm, different from the typical triangle 2H MoS_2_ flakes. The powder has a distinct metal‐like luster (Figure 2a inset). Figure 2c gives the optical image of Na_2_S‐intercalated 1T’ MoS_2_ nanosheets, showing a strip shape. The thinnest strip's thickness is about 12.43 nm, according to the atomic force microscopy (AFM) height profile result in Figure 2d. The as‐synthesized sample shows a unique Raman spectrum (Figure 2e) different from 2H MoS_2_. The characteristic peaks of 1T’ phase, *J*_1_ and *J*_3_, are distinguishing, confirming the existence of 1T’ phase MoS_2_ with high‐crystal quality. The defect‐related Raman peak *J*_2_[^7^, ^16^, ^20^] cannot be detected in these samples, indicating that there are no typical defects caused by previous solution‐phase intercalation methods. Except for characteristic peaks of 1T’ phase, there are another three Raman peaks (marked as A, B, and C) that relate to the intercalation. The as‐synthesized nanosheets and powder samples have similar Raman peaks (Figure 2e), indicating the 1T’ phase MoS_2_ structure. The n‐butyllithium treatment, commonly used for 1T’ MoS_2_ preparation,^[1b]^ was also utilized to transform the 2H‐phase MoS_2_ to 1T’ phase for comparison. Supplementary Note 1 gives the details of the preparation process. The Raman spectrum of the sample is shown in Figure 2e, and its Raman peaks are clearly weaker than those of this work's samples, suggesting a poor crystal quality.

XRD was used to investigate the sample's structure. As shown in Figure 2f, the as‐synthesized K_2_S‐intercalated 1T’ MoS_2_ has an extended interlayer distance, d_(200)_ (0.9064 nm), compared with intrinsic 1T’ MoS_2_ (0.5876 nm) as well as 2 H MoS_2_ (0.6148 nm). The intercalation causes the extension of lattice constant *a*, from 1.2835 nm to 1.9797 nm. The lattice schematic in Figure S2 (Supporting Information) illustrates the extension of interlayer distance and lattice constant *a* caused by intercalation. The clear XRD peaks with a lattice plane parallel to the basal plane of MoS_2_ prove that it prefers the growth to extend basic plane, the so‐called 2D growth. The flake‐like shape shown in Figure 2a,b also suggests this growth tendency. To fully study the lattice structure of the sample, the K_2_S‐intercalated 1T’ MoS_2_ powder was scraped off the substrate and then spread and filled in an XRD powder sample holder, and then the XRD test was conducted. This XRD result (Figure 2g) gives abundant peaks compared with the last XRD test result shown in Figure 2f, because the above process randomly rotated the orientation of MoS_2_ flakes. As the lattice constant *a* increases, the XRD peaks’ positions would greatly differ from the intrinsic 1T’ MoS_2_. Here, a lattice model (shown in Figure S2, Supporting Information) is built to simulate the XRD spectrum, in which the basal plane structure is the same as 1T’ MoS_2_, but with extended *a*. As shown in Figure 2g, the experimental XRD result is highly in accord with the simulation. These results strongly indicate that the as‐synthesized sample has the same in‐plane structure with intrinsic 1T’ MoS_2_, but with extended interlayer distance caused by intercalation.

The XPS is commonly used to evaluate the phase purity of 1T’ TMDs sample.[^1^, ^3^, ^20^] As shown in Figure 2h, the Mo 3*d* XPS spectrum shows that both Mo 3*d*_5/2_ and Mo 3*d*_3/2_ peaks have lower binding energy (228.00 and 231.17 eV) compared with 2H MoS_2_. And the binding energy is nearly the same as the reported 1T’ MoS_2_.^[20a]^ There is only one set of Mo 3*d* XPS peaks in fitting result, and the fitting curve perfectly fits the experimental curve. This confirms that the prepared sample has very high phase purity. Another three XPS tests were conducted to confirm the phase purity, as shown in Figure S4 (Supporting Information). All XPS results are very similar, with only one set of Mo 3*d* peaks in each spectrum. With the accuracy of XPS, it is determined to be very close to 100% phase pure. The existence of potassium is confirmed by K 2p XPS peaks (Figure 2i) with the binding energy of 295.0 eV (K 2*p*_1/2_) and 292.2 eV (K 2*p*_3/2_), close to the binding energy of univalent potassium K^+^. The sulfur XPS result shows two sets of fitting curves, corresponding to two different chemical environments. As shown in Figure 2j, the larger part of sulfur is from 1T’ MoS_2_, as it has the same binding energy as S of 1T’ MoS_2_, and 1:2 atom ratio of Mo and S is obtained by counting the sulfur atom amount of this part. The rest of the sulfur has higher binding energy (162.37 eV, S2*p*_3/2_) which could bond with potassium atoms, and a 2:1 atom ratio of K and S is obtained by calculating this part's sulfur atom amount. So the chemical formula is (K_2_S)_*x*_MoS_2_, with *x* = 0.18. As the Raman spectrum and XRD result prove that the in‐plane structure is the same as intrinsic 1T’ MoS_2_, the K_2_S should be in the space between layers—in other words, it is intercalated in layered 1T’ MoS_2_. The following TEM characterization provides more evidence.

**Figure 3:**
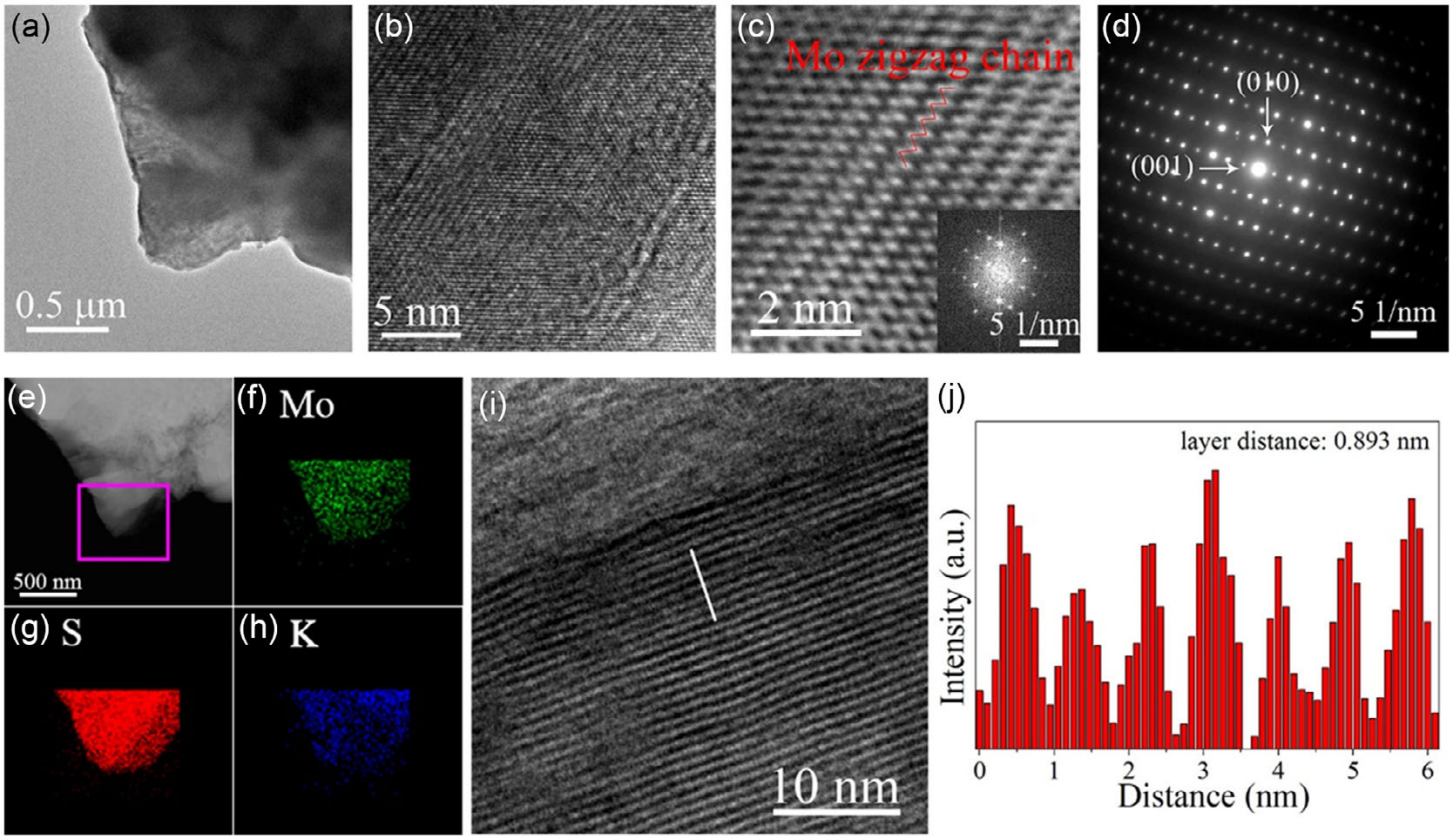
TEM characterization of K_2_S‐intercalated 1T’ MoS_2_. a,b) TEM and high‐resolution transmission electron microscopy (HRTEM) images of K_2_S‐intercalated 1T’ MoS_2_, respectively. c) FFT filtered images of (b). Inset of (c), FFT pattern of (b). d) SAED pattern of K_2_S‐intercalated 1T’ MoS_2_. e) Dark‐field STEM image of K_2_S‐intercalated 1T’ MoS_2_. f–h) Elemental mapping images of Mo, S, and K, respectively, acquired from pink rectangular box in (e). i) High‐resolution cross‐sectional TEM image. j) Brightness profile along the white line in (i).

TEM characterization is conducted to investigate the samples’ lattice structure, the results of which are given in **Figure** **3**a–d. The distinct feature of the 1T’ phase superstructure, Mo zigzag chains, can be seen in the high‐resolution TEM image (marked in Figure 3c). The schematic of the in‐plane lattice structure of 1T’ MoS_2_ (Figure S3, Supporting Information) represents the same Mo zigzag chains. The selected area electron diffraction (SAED) pattern (Figure 3d) and the fast Fourier transform (FFT) pattern (Figure 3c inset) confirm the distorted octahedral coordinated structure of 1T’ MoS_2_. As shown in Figure 3e–h, the EDS mapping result gives evidence of the existence of the Mo and S element, as well as K element which comes from the intercalation of “K_2_S”. Cross‐sectional TEM was conducted to study the intercalation further, and the results are shown in Figure 3i,j. The cross‐sectional TEM sample preparation details can be found in Supplementary Note 2. The interlayer distance is extended to 0.893 nm, which is also close to d_(200)_ (0.9064 nm) measured by XRD. The EDS (energy‐dispersive X‐ray spectroscopy) mapping images under high‐angle annular dark‐field scanning transmission electron microscopy (STEM) are given in Figure S5 (Supporting Information). The elements Mo, S, and K represent layer distribution, and each K layer is in between the Mo layers. These results further confirm that the as‐synthesized sample has 1T’ phase MoS_2_ in‐plane structure, and the “K_2_S” was intercalated between the MoS_2_ layers. The van der Waals gap is extended to 0.893 nm, which is shown in cross‐sectional TEM image and its brightness profile (Figure 3i,j). The XRD spectrum given in Figure 2f also indicates the similar extended van der Waals gap distance. The intercalation of K_2_S increases the *a*‐axis lattice parameter, from 1.2835 to 1.9797 nm.

To investigate the influence of reaction temperature on phase structure, the samples were grown at 550, 650, 750, 850, and 950 °C while keeping other parameters same. The Raman spectra of products were given in Figure S6 (Supporting Information). The sample with growth temperature of 750 °C has strongest 1T’ phase characteristic Raman peak, *J*_1_. With the decrease in growth temperature, *J*_1_ peak disappears, and the 2H phase characteristic Raman peak can be observed at 550 °C. The low‐temperature reaction cannot provide K_2_S enough energy to intercalate into the layered MoS_2_, thus leading to 2H phase formation. With the increase in growth temperature, the intensity of *J*_1_ Raman peak drops, and the product is the mixture of 1T’ and 2H phase MoS_2_ at 950 °C. The potassium sulfide melts at 840 °C, and the intercalated K_2_S would have a similar property with potassium sulfide. The temperature above 850 °C could release K_2_S from interlayer of MoS_2_ by melting or vaporization, which would destroy the intercalation structure and cause formation of 2H phase, thus lowering phase purity. Overall, 750 °C is most suitable for K_2_S‐intercalated 1T’ MoS_2_ growth.

### 2.3 Stability of Intercalated 1T’ MoS_2_

**Figure 4:**
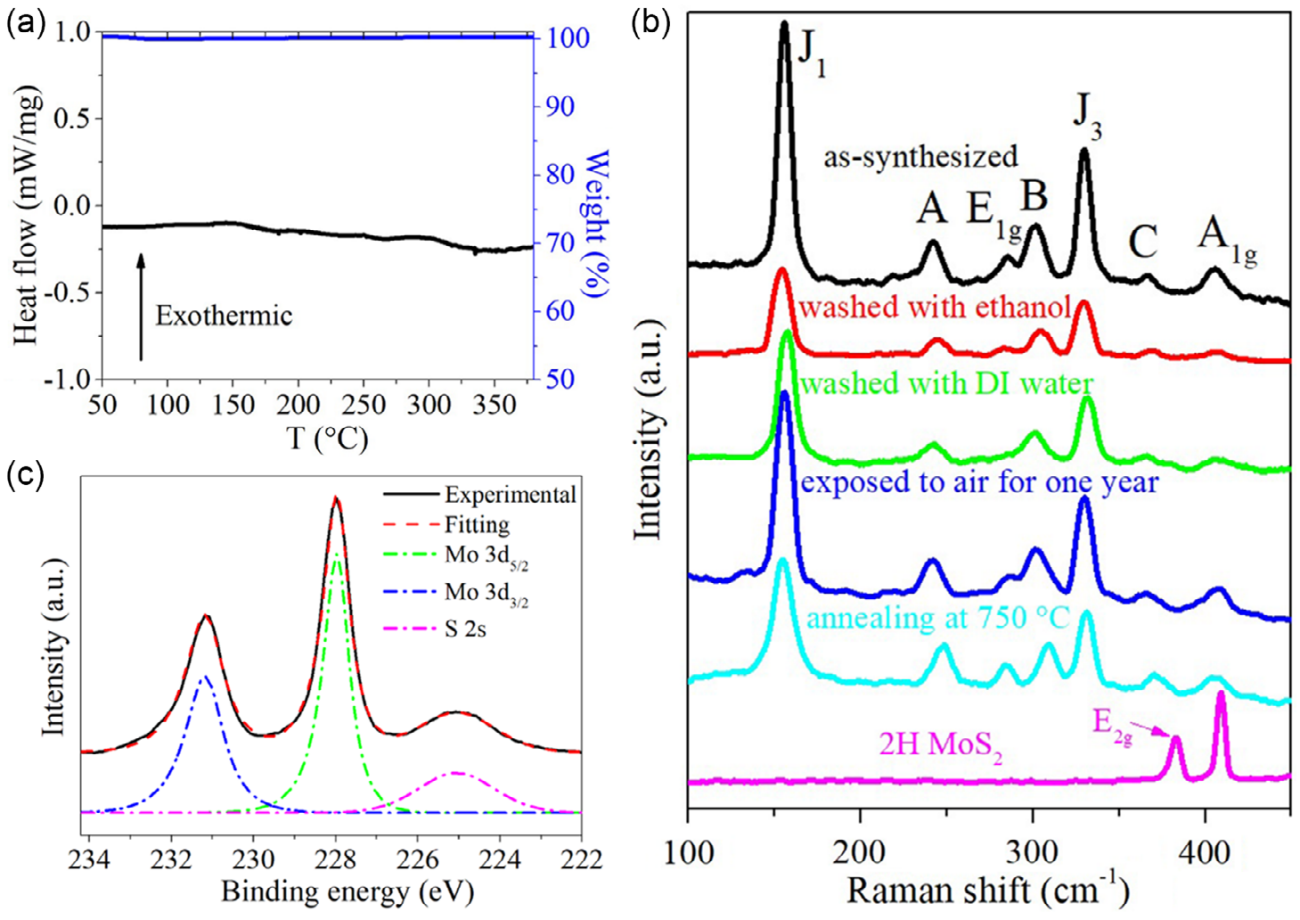
Stability of K_2_S‐intercalated 1T’ MoS_2_. a) TGA and DSC curves of K_2_S‐intercalated 1T’ MoS_2_ powder. b) Raman spectra of as‐synthesized K_2_S‐intercalated 1T’ MoS_2_ and K_2_S‐intercalated 1T’ MoS_2_ washed with ethanol, washed with DI water, exposed to air for 8 months, and after annealed at 750 °C, and 2H MoS_2_. c) Experimental and fitted XPS Mo 3*d* and S 2*s* spectra of K_2_S‐intercalated 1T’ MoS_2_ powder after annealing at 750 °C.

The as‐synthesized K_2_S‐intercalated 1T’ MoS_2_ has excellent stability in this work. The prepared sample shows resistivity against high temperature. The DSC test with temperature range from 50 to 380 °C confirmed that the K_2_S‐intercalated 1T’ MoS_2_ does not change its phase, as shown in **Figure** **4**a. And it did not lose its weight either, indicated by the TGA curve (Figure 4a). However, the intrinsic 1T’ MoS_2_ has a transition temperature as low as 97 °C,^[16a]^ at which 1T’ MoS_2_ transforms to 2H phase completely. The K_2_S‐intercalated 1T’ MoS_2_ also shows stability against solvent. The as‐prepared samples were completely washed with DI water and ethanol. There is no significant change in Raman spectra (Figure 4b). The EDS mapping (Figure S7, Supporting Information) shows the potassium still exists after washing. This indicates that the intercalated K_2_S cannot be simply considered potassium sulfide which is soluble in water and ethanol. For comparison, the Raman spectrum of commercial 2H MoS_2_ powder is also shown in Figure 4b, in which the 2H phase feature Raman peak *E*_2g_ is marked. To test the aging stability, the as‐synthesized samples are exposed to air at room temperature for 1 year, and there is no sign of degradation, as shown in the Raman spectrum in Figure 4b. The samples show excellent stability, different from the 1T’ MoS_2_ prepared by other methods,^[^ ^3^, ^16^, ^20^, ^21^ ^]^ thus making it promising in various practical applications. To further investigate the thermal stability of sample, the as‐synthesized K_2_S‐intercalated 1T’ MoS_2_ was annealed at 750 °C just after the synthesis process, and then it was characterized by Raman spectroscopy and XPS. The Raman spectrum (Figure 4b) shows no apparent change compared with the as‐synthesized sample. The XPS spectrum of Mo 3*d* and S 2*s* in Figure 4c further confirms that the sample keeps its 100% 1T’ phase purity even after 750 °C annealing. The above results indicate that K_2_S intercalation provides high stability against aging and high temperature and is inert in common environment such as air, ethanol, and water. The relationship between thickness and stability was also studied. The results and discussion can be found in Supplementary Note 3.

To investigate the influence of K_2_S intercalation further, we conducted the calculation in the framework of the DFT, the details can be found in Supplementary Note 4. To evaluate the influence of K_2_S intercalation on the stability of MoS_2_ with different phases, the models of K_2_S‐intercalated 1T’‐ and 2H‐MoS_2_ were constructed. The theoretical calculation results indicate that with the intercalation of K_2_S, 1T’ MoS_2_ is more energetically stable than 2H MoS_2_. This increased stability of 1T’ phase over the 2H phase is attributed primarily to the interlayer confinement effect of K_2_S intercalation and enhanced N‐doping effect in the MoS_2_.

Except for the K_2_S‐intercalated 1T’ MoS_2_, Li_2_S and Na_2_S‐intercalated 1T’ MoS_2_ are also synthesized by this method. K_2_Se‐intercalated 1T’ MoSe_2_, Na_2_Se‐intercalated 1T’ MoSe_2_, and K_2_Te‐intercalated 1T’ MoTe_2_ are all successfully prepared, indicating that this method can be commonly used among various TMDs. The details of the synthesis process are given in the Experimental Section, and the characterization results of these samples are shown in Figure S16–S24 (Supporting Information).

### 2.4 HER Performance and Long‐Term HER Stability

**Figure 5:**
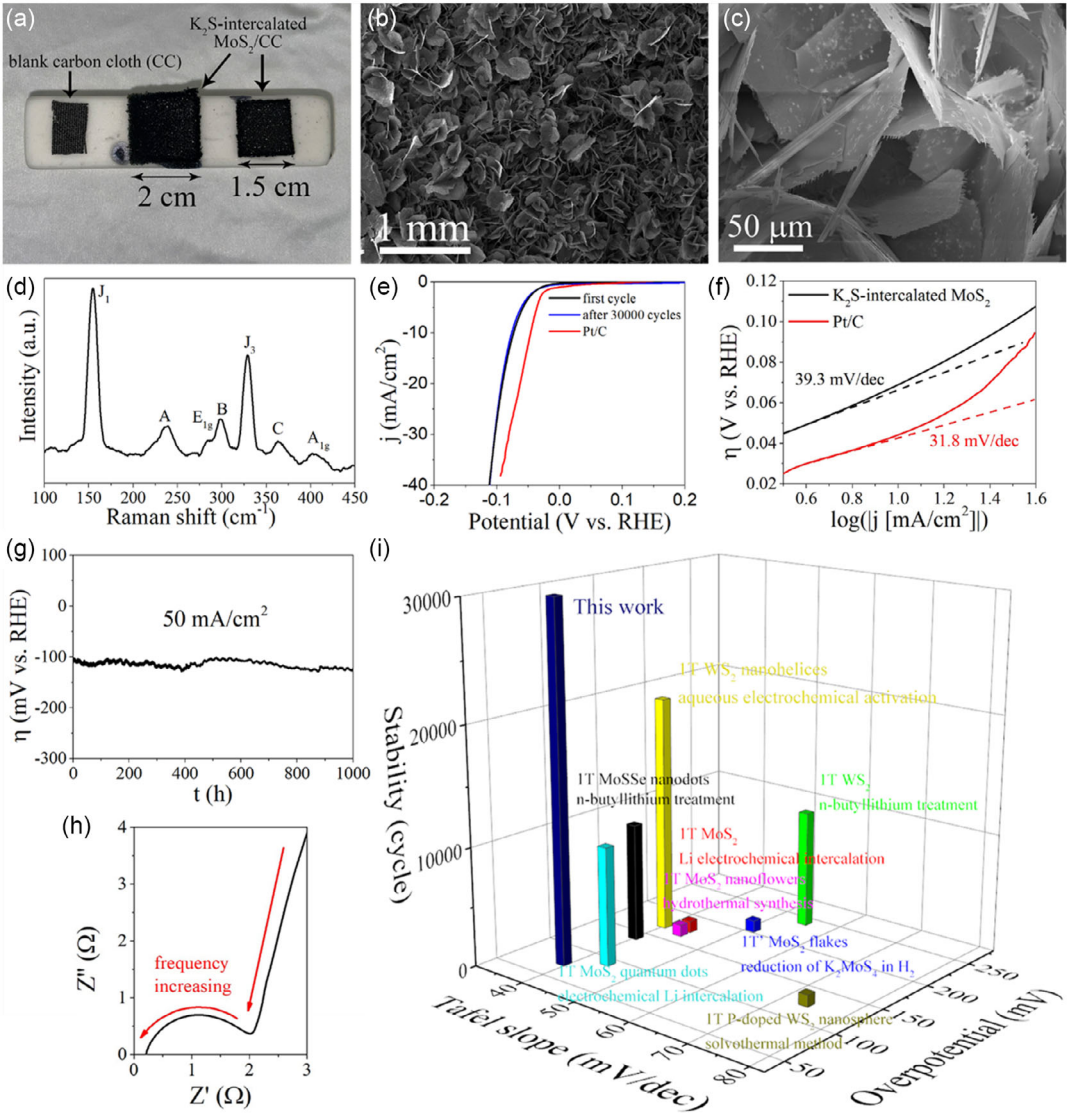
HER performance of K_2_S‐intercalated 1T’ MoS_2_. a) Photo of blank carbon cloth and carbon cloth with K_2_S‐intercalated 1T’ MoS_2_ grown on. b) SEM image of K_2_S‐intercalated 1T’ MoS_2_ grown on carbon cloth. c) Zoom‐in SEM image of K_2_S‐intercalated 1T’ MoS_2_ flakes grown on carbon cloth. d) Raman spectrum of K_2_S‐intercalated 1T’ MoS_2_ grown on carbon cloth. e) The polarization curves (iR corrected) of K_2_S‐intercalated 1T’ MoS_2_ HER electrocatalyst before and after 30 000 cycles, and commercial Pt/C HER electrocatalyst. f) The corresponding Tafel slopes of K_2_S‐intercalated 1T’ MoS_2_ and commercial Pt/C HER electrocatalysts derived from e. g) Overpotential versus time curve of K_2_S‐intercalated 1T’ MoS_2_ HER electrocatalyst with constant current density of 50 mA cm^−2^. h) Nyquist plot of K_2_S‐intercalated 1T’ MoS_2_ electrocatalyst. i) Comparison with best TMDs‐based HER electrocatalysts in three perspectives (Tafel slope, overpotential, and stability). The details of all compared cases shown in (i), including phase purity, electrode type, and literature source, are listed in Table S4 (Supporting Information).

The 1T’ MoS_2_ has been considered an excellent earth‐abundant electrocatalyst for HER, as it is metallic, which helps transport of electrons, and has high density of catalysis active sites. Both theory calculation and experimental data^[^ ^22^ ^]^ prove its advanced electrochemical HER performance. However, the intrinsic 1T’ MoS_2_ has a critical drawback; the 1T’ MoS_2_‐based electrocatalysts usually show insufficient stability as 1T’ phase is metastable. Here, an example of application, HER, benefiting from stabilization and phase purity achieved by this method is demonstrated. To conduct the HER test, the K_2_S‐intercalated 1T’ MoS_2_ was directly grown on carbon cloth, and the detailed process can be found in the Experimental Section, with a few slight changes in the proposed method. **Figure** **5**a is the photo of blank carbon cloth, and the carbon cloth with K_2_S‐intercalated 1T’ MoS_2_ grown on, which is called K_2_S‐intercalated 1T’ MoS_2_/carbon cloth for simplification in this article. As shown in scanning electron microscope (SEM) images (Figure 5b,c), the intercalated 1T’ MoS_2_ flakes stand on carbon cloth's fibers that were fully covered.

An electrochemical configuration with H‐type cell was employed in HER measurements, which was carefully designed to avoid contaminations from reference electrode and counter electrode; more details can be found in the Experimental Section and Supplementary Note 5. The electrochemical HER performance of commercially available Pt/C was also tested for comparison. Not like many previously reported 1T’ TMDs‐based electrocatalysts, which are covered by Nafion film,[^7^, ^13^, ^23^] the K_2_S‐intercalated 1T’ MoS_2_/carbon cloth can be directly immersed in 0.5 m H_2_SO_4_ electrolyte during HER test, showing stability against strong acid. The polarization curves (iR corrected) of K_2_S‐intercalated 1T’ MoS_2_ and commercially available Pt/C are shown in Figure 5e. A low onset potential of −73 mV for K_2_S‐intercalated 1T’ MoS_2_ was obtained at current density of 10 mA cm^−2^. The corresponding Tafel slope is 39.3 mV dec^−1^, as shown in Figure 5f. Importantly, the K_2_S‐intercalated 1T’ MoS_2_ shows excellent long‐term stability. Even after 30 000 cyclic voltammetry (CV) cycles (overpotential range: −0.25–0.2 V vs. RHE), there is no significant change in polarization curve (Figure 5e). The SEM image of sample after 30 000 cycles (Figure S25, Supporting Information) shows the K_2_S‐intercalated 1T’ MoS_2_ flakes still stand on the carbon cloth; there is no significant morphology change. Raman spectrum (Figure S26, Supporting Information) also confirms the sample keeps 1T’ phase after long‐term stability test. To fully and accurately investigate the long‐term stability, chronopotentiometric analysis was also conducted. Using the above‐described electrochemical configuration, the prepared K_2_S‐intercalated 1T’ MoS_2_ had acted as HER electrocatalyst for 1000 h at a constant current density of 50 mA cm^−2^. The overpotential was recorded constantly, the overpotential versus time curve (Figure 5g) shows minimal fluctuation in this 1000‐hour period and no degeneration trend. The Figure 5h is a Nyquist plot of K_2_S‐intercalated 1T’ MoS_2_ electrocatalyst, with frequency from 1 Hz to 10^5^ Hz. The charge transfer resistance *R*_ct_ is as low as 1.8 Ω, thanks to the highly HER‐active metallic 1T’ MoS_2_. It is confirmed that the K_2_S‐intercalated 1T’ MoS_2_ possesses excellent electrochemical HER performance (onset potential of −73 mV at 10 mA cm^−2^ and Tafel slope of 39.3 mV dec^−1^), comparable with the best TMDs‐based HER electrocatalysts and nonprecious HER electrocatalysts, and notably its 1000‐hour stability at a current density of 50 mA cm^−2^ and 30 000‐cycle stability make it stand out among counterparts. The comparison of reported electrocatalysts for HER is listed in Table S4 and S5 (Supporting Information). Figure 5i demonstrates the comparison between K_2_S‐intercalated 1T’ MoS_2_ and best TMDs‐based HER electrocatalysts considering three critical factors, Tafel slope, overpotential, and stability. It shows that the K_2_S‐intercalated 1T’ MoS_2_ synthesized by this work possesses outstanding HER performance.

The excellent HER performance is mainly due to high phase purity. The 2D 1T’ MoS_2_ flakes have catalysis active sites at the edge and on the plane, while 2D 2H MoS_2_ flakes only have them at edge.^[^ ^22^ ^]^ The upper and lower surfaces of layered MoS_2_ flakes are regarded as basal plane. This property of 1T’ MoS_2_ can greatly increase the density of HER active sites. The metallic 1T’ phase also boosts the transport of electrons, but semiconducting 2H MoS_2_ definitely blocks it. Therefore, the phase‐pure 1T’ MoS_2_ prepared by this work shows low onset potential and low Tafel slope. Moreover, the K_2_S intercalation provides stability and prevents it from phase transition, while the intrinsic 1T’ phase transforms to 2H phase over time, and it is vulnerable during HER, thus overcoming the biggest drawback of 1T’ MoS_2_. As a result, the superb long‐term stability of HER is obtained.

## 3 Conclusions

In summary, a mass‐production‐available method involving self‐intercalation has been developed to synthesize phase‐pure K_2_S‐intercalated 1T’ MoS_2_ with excellent stability against aging (over year) and high temperature (over 750 °C). In comparison, intrinsic 1T’ MoS_2_ is metastable and has a transition temperature as low as 97 °C and even degenerates over time at room temperature. This method supports mass production, as it uses cheap reactants and equipment, and only requires one‐step process for a few hours. Moreover, this method can be commonly used among various TMDs. Except the K_2_S‐intercalated 1T’ MoS_2_, the Li_2_S‐intercalated and Na_2_S‐intercalated 1T’ MoS_2_ are also synthesized by this method. The K_2_Se‐intercalated 1T’ MoSe_2_, Na_2_Se‐intercalated 1T’ MoSe_2_, and K_2_Te‐intercalated 1T’ MoTe_2_ are all successfully prepared too, indicating versatility of this method. As an example of application benefiting from stabilization and phase purity, K_2_S‐intercalated 1T’ MoS_2_ nanoflakes were synthesized and acted as a HER electrocatalyst. It shows excellent HER performance with a low onset potential of −73 mV at the current density of 10 mA cm^−2^ and low Tafel slope of 39.3 mV dec^−1^, comparable with the best non‐precious HER electrocatalysts including TMDs, due to the phase purity. Importantly, the K_2_S intercalation brings much better HER long‐term stability (30 000 cycles and 1000 h with constant current density of 50 mA cm^−2^) compared with its counterparts. This work opens up a new route to synthesize phase‐pure 1T’ TMDs and stabilize them via intercalation without changing the in‐plane structure. It enables the more accessible investigation of novel properties of metastable phase TMDs and development of their promising various applications.

## 4 Experimental Section

4.1

4.1.1

##### Synthesis of K_*2*_ *S‐Intercalated 1T’ MoS*_*2*_

A three‐heating‐zone furnace with a 1‐m‐long quartz tube was used to directly synthesize 1T’ MoS_2_, as shown in Figure S1 (Supporting Information). The quartz tube was completely clean, and there were no any remaining substances allowed, especially sulfur which usually remained if reusing quartz tube. In this work, a new and clean quartz tube was used in each synthesis. Sulfur powder (10 g) was loaded into an alumina boat, which was put in left heating zone. The sulfur was excess reagent and fully covered the bottom of cuboid alumina boat (bottom area: 910 mm^2^) throughout the whole synthesis process to obtain a uniform supply of sulfur by evaporation. K_2_MoO_4_ powder (60 mg) was placed in the right heating zone. The sapphire substrate with an area of 2 × 2 cm^2^ was used to support K_2_MoO_4_ powder. And the substrate was put on an alumina boat in right heating zone. The K_2_MoO_4_ powder was fully spread and exposed. The middle heating zone was left empty. After all materials were loaded, the tube was pumped to eliminate the air inside, and then the argon gas was flowed into it until atmospheric pressure was reached. Next, the argon flow was kept at 35 sccm, and hydrogen gas was also flowed into the tube at 15 sccm. The synthesis was conducted at atmospheric pressure. The middle and right heating zone were heated to 750 °C at a rate of 15 °C min^−1^, as left heating zone remained at room temperature. Once these two heating zones reached growth temperature, 750 °C, the left heating zone started to go to 135 °C at a heating rate of 30 °C min^−1^. This heating process ensured there was no sulfur vapor before right and middle zone reached growth temperature (750 °C); thus, no reaction occurred at temperature lower than 750 °C. It is worth noticing that the quartz tube, if it is reused, should be cleaned entirely before synthesis. The remaining sulfur vaporizes during heating and low‐temperature reaction occurs, which must be avoided. The middle and right heating zone was kept at 750 °C for 4 h and then cooled down to room temperature naturally; once these two zones started to cool down, the power of left heating zone was also shut down and cooled down naturally. The sample was taken out after furnace had been completely cooled down, and the K_2_S‐intercalated 1T’ MoS_2_ was obtained without any post‐treatment.

##### Deposition of Na_*2*_ *MoO*_*4*_ *Film and Na*_*2*_ *S‐intercalated 1T’ MoS*_*2*_ *Nanosheet Synthesis*

Na_2_MoO_4_ film was grown on a sapphire substrate by sputtering deposition (sputtering target: Na_2_MoO_4_, 50 W, 600 s, and 20 sccm Ar/10 sccm O_2_). Then, the Na_2_MoO_4_ film replaced the K_2_MoO_4_ powder, and the other process steps were same as the above‐described K_2_S‐intercalated 1T’ MoS_2_ synthesis. After that, the Na_2_S‐intercalated 1T’ MoS_2_ nanosheets on sapphire substrate were obtained.

##### Synthesis of Na_2_S‐Intercalated and Li_2_S‐Intercalated 1T’ MoS_2_

It is very similar to the synthesis of K_2_S‐intercalated 1T’ MoS_2_, just replace K_2_MoO_4_ with Na_2_MoO_4_ or Li_2_MoO_4_ for Na_2_S‐intercalated or Li_2_S‐intercalated 1T’ MoS_2_ synthesis. The growth temperature is 650 °C for Na_2_S‐intercalated 1T’ MoS_2_ and is 750 °C for Li_2_S‐intercalated 1T’ MoS_2_.

##### Synthesis of Na_2_Se‐intercalated and K_2_Se‐intercalated 1T’ MoSe_2_

It was highly similar to the synthesis of Na_2_S‐intercalated and K_2_S‐intercalated 1T’ MoS_2_ described above. The sulfur powder was replaced with selenium powder, and the heating temperature was 230 °C. For the synthesis of Na_2_Se‐intercalated 1T’ MoSe_2_, the growth temperature was 750 °C.

##### Synthesis of K_2_Te‐Intercalated 1T’ MoTe_2_

It was almost the same as the synthesis of K_2_S‐intercalated 1T’ MoS_2_. Sulfur powder was replaced with tellurium powder, of which the heating temperature was 500 °C.

##### Preparation of K_2_S‐Intercalated 1T’ Mo_2_2/Carbon Cloth

First, the carbon cloth was put on 130 °C hotplate, and few drops of ethanol were dropped on it to soak the carbon cloth. Then, 10 drops of saturated K_2_MoO_4_ solution were dropped on the carbon cloth one by one in 1 min. Next, the carbon cloth was left on 130 °C hotplate for 20 min to dry. After that, the solid K_2_MoO_4_ was uniformly attached to the carbon cloth. The following process was the same as the synthesis of K_2_S‐intercalated 1T’ MoS_2_, except the K_2_MoO_4_/carbon cloth replaced the K_2_MoO_4_ powder/sapphire. The loading mass was 22.7 mg cm^−2^.

##### HER Measurements

An electrochemical configuration with H‐type cell was employed in HER measurements, with electrolyte of 0.5 m H_2_SO_4_ solution, a working electrode of K_2_S‐intercalated 1T’ MoS_2_/carbon cloth, a saturated Ag/AgCl reference electrode, and a graphite counter electrode. The H‐type cell consisted of two small cells; they are separated by a piece of Nafion film. The graphite counter electrode was placed in one cell, and the working electrode and reference electrode were placed in the other cell. A double bridge structure with Vycor glass junctions was used to set saturated Ag/AgCl reference electrode. The electrochemical HER performance of commercially available Pt/C was also tested for comparison. Similar to the commonly used method, the Pt/C was drop‐dried on glassy carbon and then covered with Nafion film.

##### Characterizations

The optical pictures were taken with Leica DM2700 optical microscopy. SEM images were acquired with Tescan MAIA3 field emission SEM. AFM images and height profiles were taken with Asylum MFP‐3D Infinity scanning probe microscope. Raman spectra were collected with WITEC Confocal Raman spectroscopy, using a 532 nm laser excitation with the power of 0.5 mW. XRD patterns were obtained with Rigaku SmartLab X‐ray diffractometer, equipped with 9 kW rotating anode X‐ray source with scintillation counter and 1D high‐speed detector. XPS spectra were captured with Thermo Fisher Scientific Nexsa XPS equipped with monochromatic and focused 12 kV Aluminum Kα X‐ray. TEM images and EDS mapping were obtained with Thermo Fisher Spectra 300 scanning transmission electron microscopy. The DSC curve was taken with Mettler Toledo differential scanning calorimeter with temperature range from −90 to 400 °C and heating/cooling rates from 0.1 to 300 °C min^−1^. The TGA curve was obtained with Mettler Toledo TGA/DSC3+ (balance sensitivity: 0.01 μg).

##### DFT Calculations

The DFT calculations were mainly conducted in Cambridge Serial Total Energy Package (CASTEP).^[^ ^24^ ^]^ The generalized gradient approximation‐Perdew Burke Ernzerhof (GGA‐PBE) functional^[^ ^24^ ^]^ was used. The 500 eV of cutoff energy was set for plane wave. The self‐consistent field (SCF) tolerance was 10^−5^ eV. The k‐point interval of 0.07(1/Å) was used under Γ‐centered sampling (unless otherwise specified). The max SCF cycle was 5000 for the calculation of band diagrams, intercalation energies, and partial density of states. Geometric optimization at GGA‐PBE level is employed to relax the atomic coordinate to the ground state with the convergence tolerance of peak force less than 0.01 eV Å^−1^. The intercalation energies of K_2_S intercalated 1T’ MoS_2_ calculated by Vienna Ab initio Simulation Package (VASP) and CASTEP are compared. The models were constructed based on the relative stoichiometry measured by XPS.

## Conflict of Interest

The authors declare no conflict of interest.

## Author Contributions

**Zongliang Guo**: conceptualization (lead); data curation (equal); formal analysis (equal); investigation (lead); methodology (lead); validation (equal); visualization (equal); writing—original draft (equal); and writing—review and editing (equal). **Hao Cheng**: data curation (supporting); formal analysis (equal); investigation (supporting); methodology (equal); software (lead); validation (equal); and writing—original draft (supporting). **Ming Yang**: conceptualization (supporting); data curation (supporting); formal analysis (supporting); investigation (supporting); methodology (equal); software (lead); supervision (equal); and validation (equal). **Chi Ho Wong**: conceptualization (equal); data curation (equal); formal analysis (equal); investigation (equal); methodology (equal); validation (equal); visualization (equal); and writing—review and editing (equal). **Tawsif Ibne Alam**: data curation (supporting); formal analysis (supporting); investigation (supporting); methodology (equal); and validation (supporting). **Shu Ping Lau**: data curation (equal); formal analysis (equal); funding acquisition (lead); supervision (equal); validation (equal); and writing—review and editing (equal). **Yuen Hong Tsang**: data curation (equal); formal analysis (equal); funding acquisition (lead); project administration (lead); resources (lead); supervision (lead); validation (lead); writing—original draft (lead); and writing—review and editing (equal).

## Supporting information

Supplementary Material

## Data Availability

The data that support the findings of this study are available from the corresponding author upon reasonable request.
