# Supplementary material for: Phase‐Pure 1T’ Molybdenum Disulfide Synthesis and Stabilization
Source: Small Sci. 2025 Mar 21;5(7):2500107. doi: 10.1002/smsc.202500107 (PMC12257880; doi:10.1002/smsc.202500107)
Supplement: Supplementary file 1 — Supplementary Material [file SMSC-5-2500107-s001.pdf]

Supporting Information

Phase-pure 1T’ Molybdenum Disulfide Synthesis and Stabilization

Zongliang Guo, Hao Cheng, Ming Yang, Chi Ho Wong, Tawsif Ibne Alam, Shu Ping Lau, and Yuen Hong Tsang*

Z. L. Guo, H. Cheng, M. Yang, T. I. Alam, S. P. Lau, Y. H. Tsang
Department of Applied Physics
The Hong Kong Polytechnic University
11 Yuk Choi Rd, Hung Hom, Kowloon, Hong Kong SAR, China
E-mail: yuen.tsang@polyu.edu.hk

Z. L. Guo, T. I. Alam, Y. H. Tsang
Shenzhen Research Institute
The Hong Kong Polytechnic University
Shenzhen, Guangdong, 518057, China

Z. L. Guo, T. I. Alam, Y. H. Tsang
Photonic Research Institute
The Hong Kong Polytechnic University
11 Yuk Choi Rd, Hung Hom, Kowloon, Hong Kong SAR, China

Z. L. Guo, T. I. Alam, Y. H. Tsang
Research Institute for Advanced Manufacturing
The Hong Kong Polytechnic University
11 Yuk Choi Rd, Hung Hom, Kowloon, Hong Kong SAR, China

C. H. Wong
Division of Science, Engineering, and Health Studies
School of Professional Education and Executive Development
The Hong Kong Polytechnic University
11 Yuk Choi Rd, Hung Hom, Kowloon, Hong Kong SAR, China

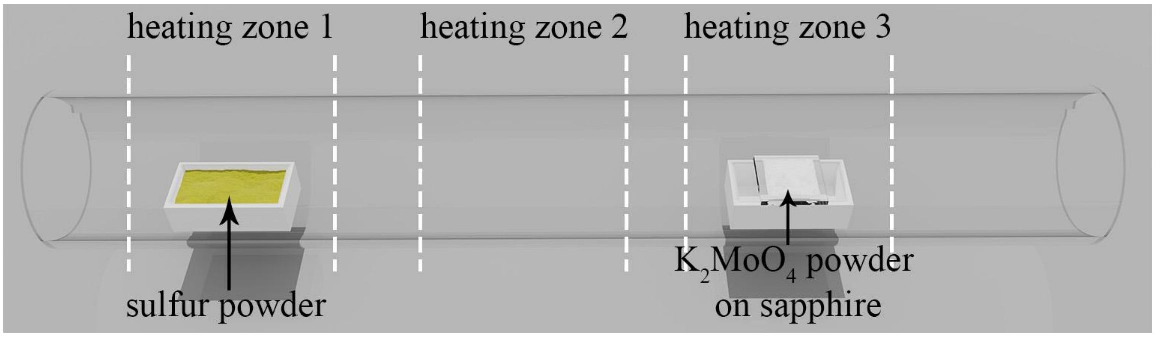

Figure S1. Schematic of K_2S-intercalated 1T' MoS_2 synthesis setup.

Supplementary Note 1

The n-butyllithium treatment was utilized to transform the 2H-phase MoS_2 flakes prepared by chemical vapor deposition (CVD) to 1T' phase for comparison. The 2H-phase MoS_2 flakes were grown on SiO_2/Si substrate by CVD using MoO_3 and sulfur powder as precursors. Then, the sample was immersed in 2 M n-Butyllithium solution in cyclohexane for 200 hours in the glove box with pure argon as protection gas. After that, the sample was completely washed with cyclohexane and dried.

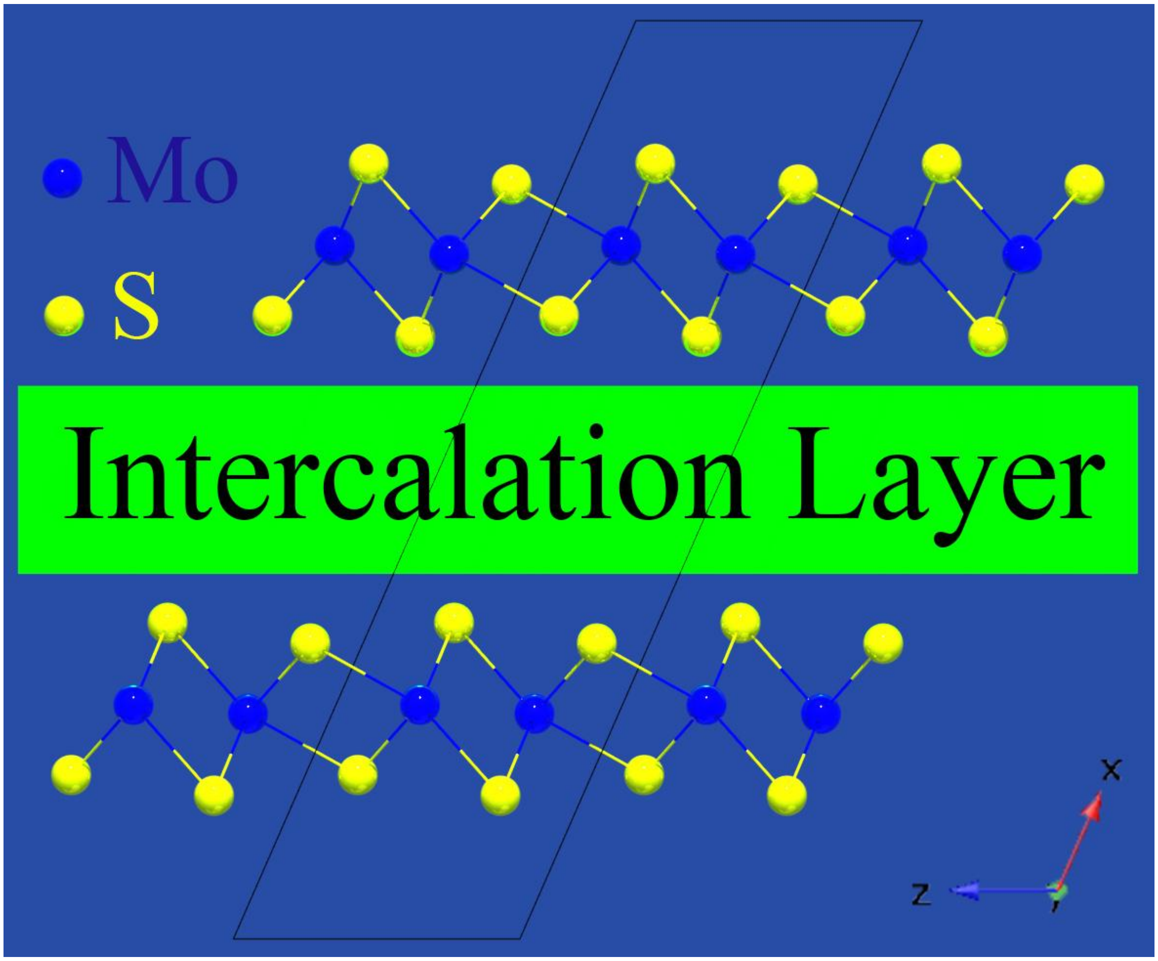

Figure S2. Lattice model of K_2S-intercalated 1T' MoS_2. Lattice constant *a*, *b*, *c* is along the direction of x, y, z axis, respectively.

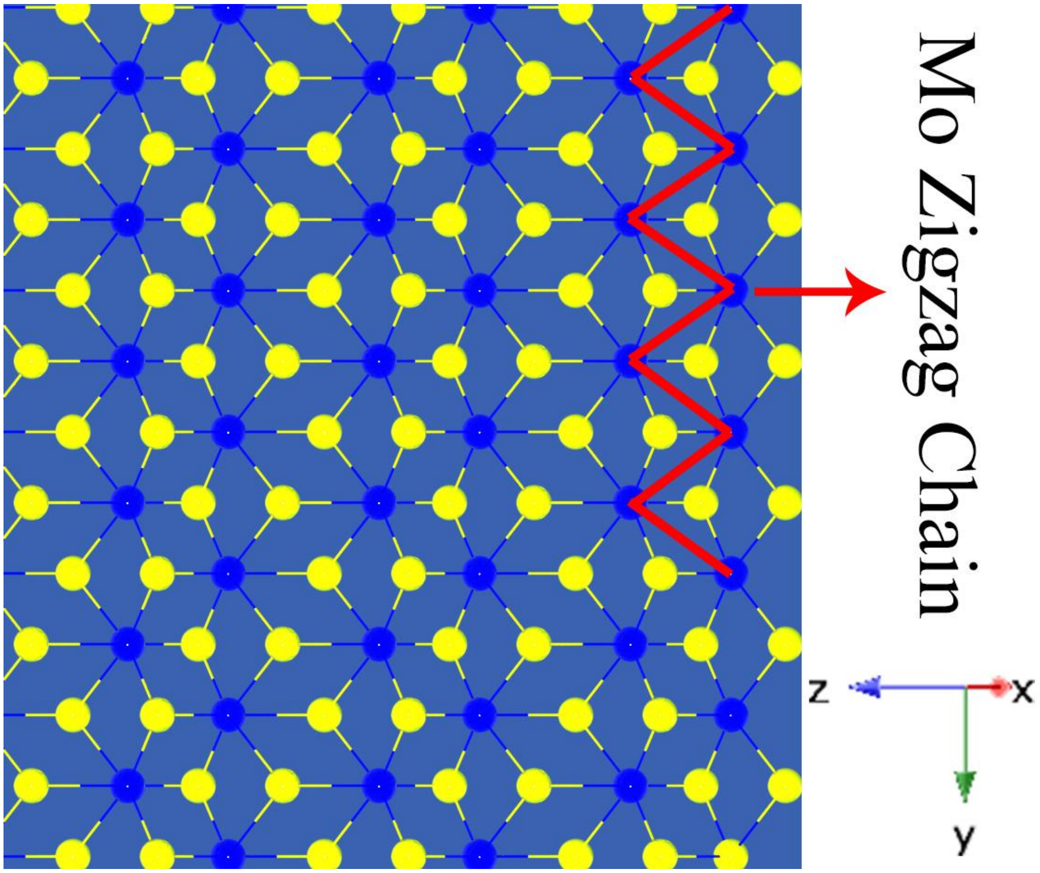

Figure S3. In-plane lattice structure of 1T' MoS_2 showing distinct Mo zigzag chains.

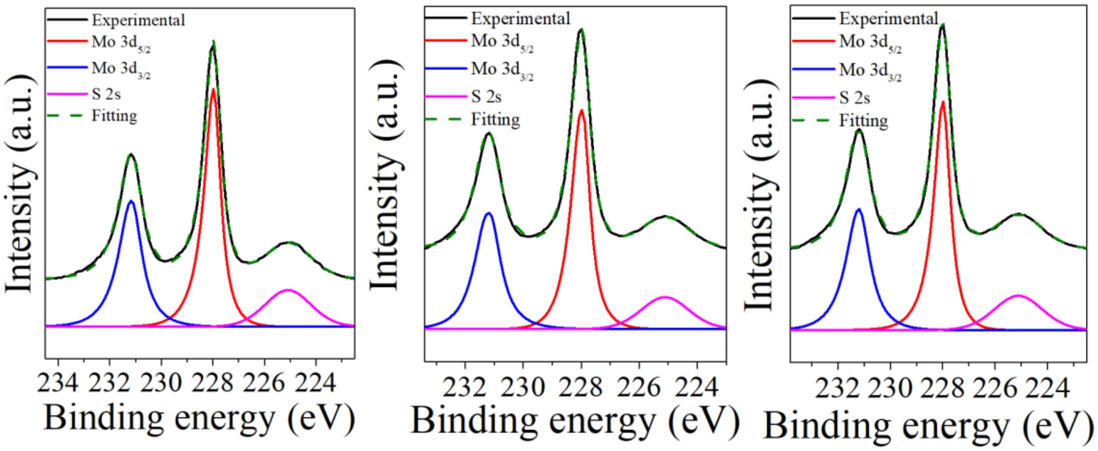

Figure S4. Another three XPS results of K_2S-intercalted 1T' MoS_2.

Supplementary Note 2

The cross-sectional TEM samples were prepared using the following method. First, the K_2S-intercalated 1T' MoS_2 was ultrasonically separated in ethanol and then drop-dried on Si substrate. Selected a piece of K_2S-intercalated 1T' MoS_2 flake and located the zone. A carbon protection layer was deposited onto the sample surface. Next, focused ion beam-scanning electron microscopy (FEI Scios) was utilized to cut the two long sides of cross-sectional TEM sample, then rotated the sample and cut the bottom and one short side. Finally, the cross-sectional TEM sample was lifted by a manipulator and welded on the holder.

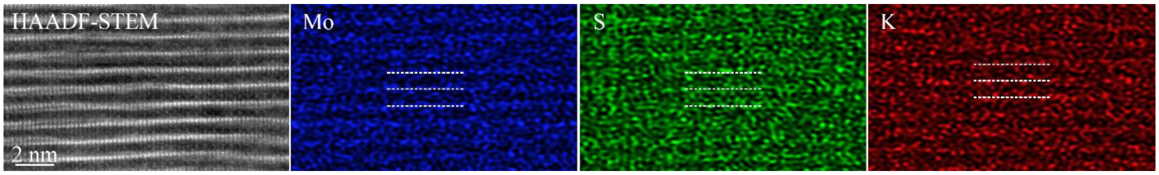

Figure S5. HAADF-STEM image and EDS mapping of Mo, S, and K.

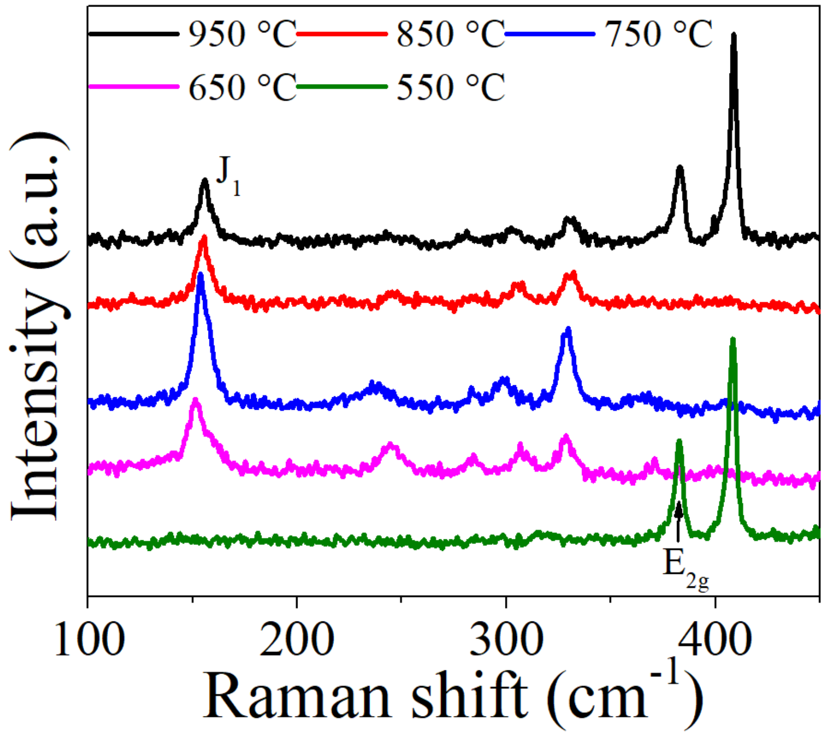

Figure S6. Raman spectra of samples with growth temperature from 550 °C to 950 °C.

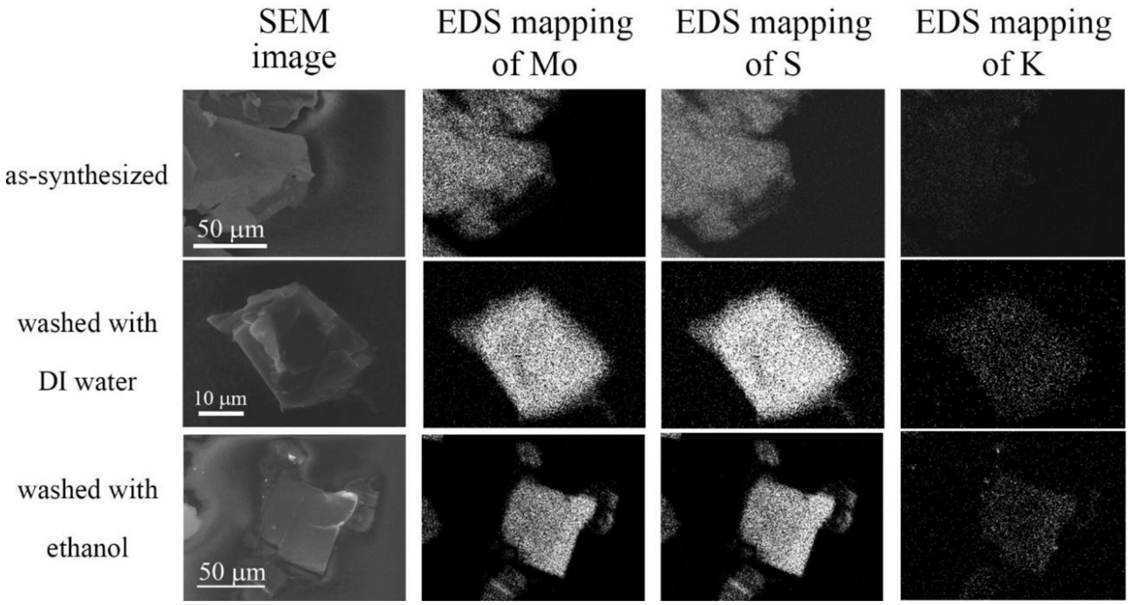

Figure S7. SEM images and EDS mapping results of as-synthesized K_2S-intercalated 1T' MoS_2 and K_2S-intercalated 1T' MoS_2 after washed with DI water and ethanol.

Supplementary Note 3

Interestingly, the intercalation of K_2S can stabilize the 1T' MoS_2, which arouses our interest in the relationship between thickness and stability, as a thicker flake consists of more intercalated K_2S and monolayer MoS_2 would have no space for intercalation. The synthesized K_2S-intercalated 1T' MoS_2 was exfoliated by tape and the thickness was measured by AFM. It is found the thinner flakes seek to be more transparent under optical microscopy. Here, two flakes with thickness of 10 nm and 34 nm were selected, and their optical images are shown in Figure S8 and Figure S10, respectively. The Raman spectra of these two flakes are given in Figure S9a and Figure S11a, both kept 1T' phase after tape exfoliation. Then they were put on a 100 °C hotplate in air for 30 min. Their Raman spectra were acquired again, as shown in Figure S9b and Figure S11b. The 10-nm-thick flake became a mix of 1T' phase and 2H phase (confirmed by appearance of E_{2g} feature Raman peak), while the flake with thickness of 34 nm showed no apparent change in Raman spectrum and kept 1T' phase. These results indicate that the stabilization against high temperature by intercalation requires the flakes to be thicker than at less 10 nm. It's found that the thinnest as-synthesized flakes obtained by this synthesis method are 12.43 nm thin, as shown in Figure 1d.

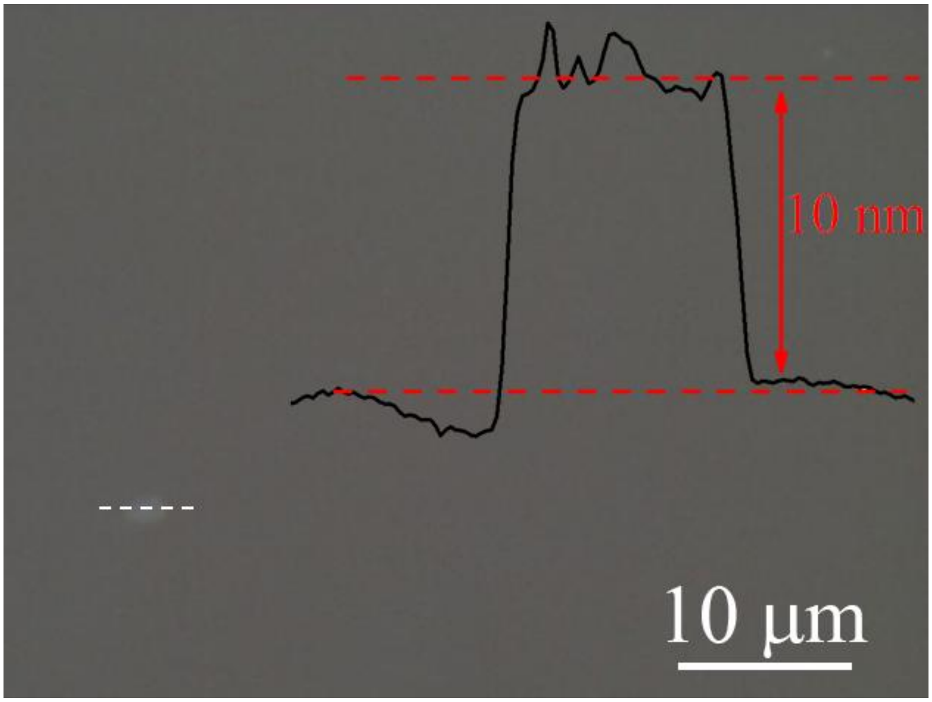

Figure S8. Optical image of 10-nm-thick K_2S-intercalated 1T' MoS_2 flake obtained by tape exfoliation and height profile along white dot line measured by AFM.

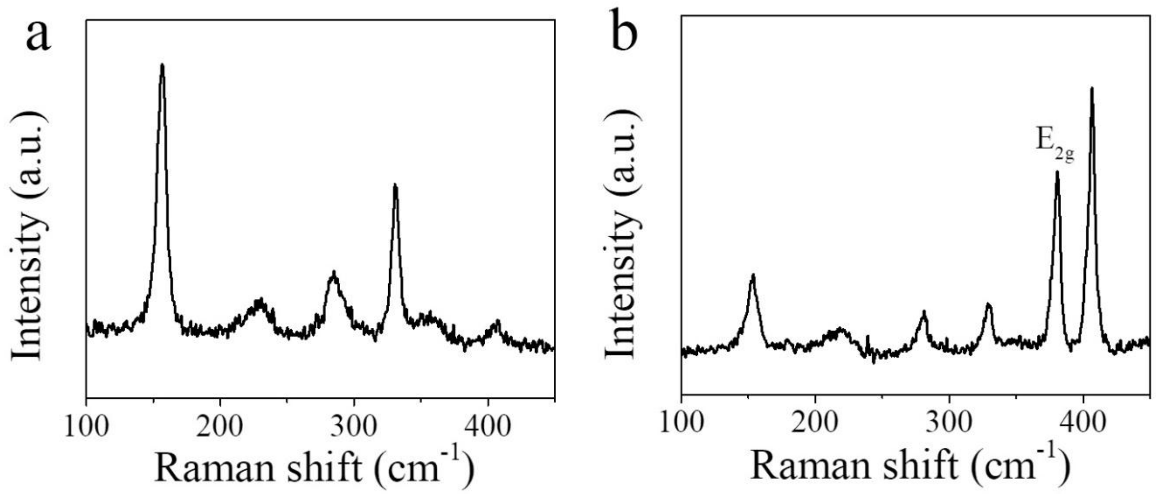

Figure S9. Raman spectra of 10-nm-thick K_2S-intercalated 1T' MoS_2 flake obtained by tape exfoliation. a, Before 100 °C annealing. b, After 100 °C annealing.

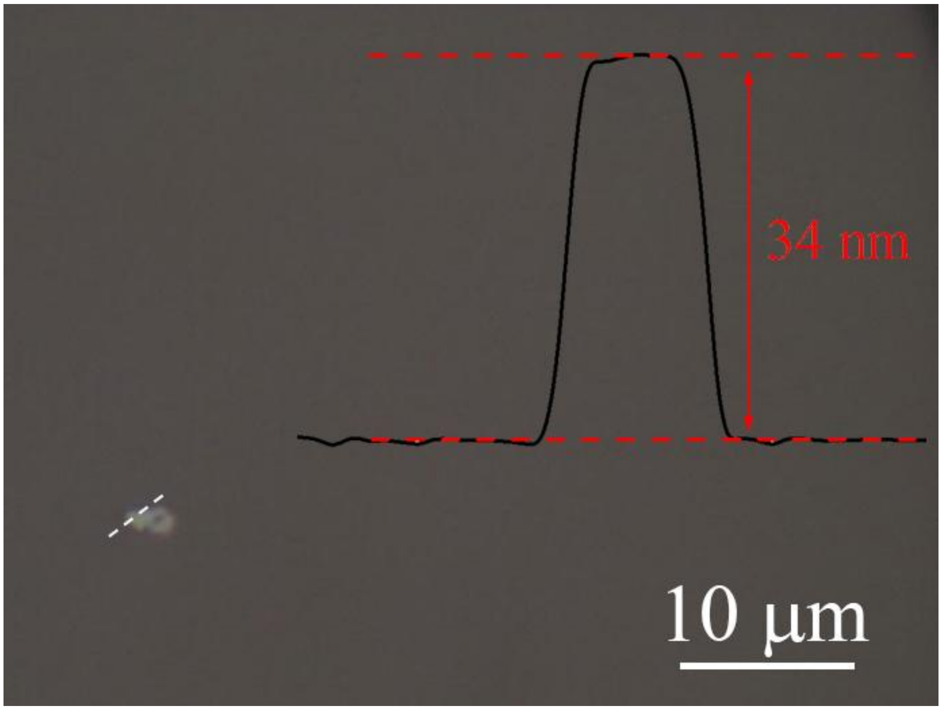

Figure S10. Optical image of 34-nm-thick K_2S-intercalated 1T' MoS_2 flake obtained by tape exfoliation and height profile along white dot line measured by AFM.

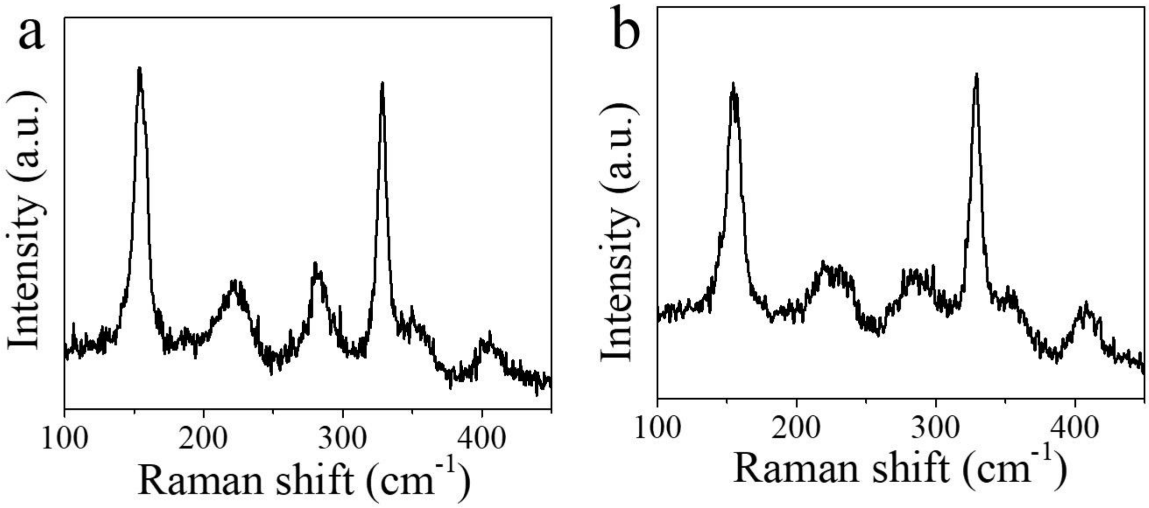

Figure S11. Raman spectra of 34-nm-thick K_2S-intercalated 1T' MoS_2 flake obtained by tape exfoliation. a, Before 100 °C annealing. b, After 100 °C annealing.

Supplementary Note 4

Density functional theory (DFT) calculations were performed using the Vienna Ab initio Simulation Package (VASP) with projector augmented wave (PAW) pseudopotentials.[1] The Perdew–Burke–Ernzerhof (PBE) functional[2] was employed to approximate the exchange-correlation interaction, utilizing a plane-wave basis set with an energy cutoff of 450 eV. Van der Waals interactions were accounted using the Grimme-D3 scheme.[3] The Fermi-level smearing width was set to 0.05 eV for both geometry optimization and electronic structure calculations. The Brillouin zone was sampled with 5×5×5 and 4×16×8 Monkhorst-Pack k-point grids for K_2S cubic bulk and 1T'-MoS_2 bulk phases, respectively. Spin polarization was incorporated into all calculations, and model structures were optimized until force components were reduced below 0.05 eV/Å. The lattice constant of K_2S cubic bulk and 1T'-MoS_2 were calculated to be 7.39 Å and a = 12.39 Å, b = 3.20 Å, c = 5.76 Å, β = 114.7°, respectively, aligning well with reference data.[4]

To examine the structural stability of K_2S-intercalated MoS_2, two intercalation models were considered: K_2S-intercalated 1T'- and 2H-MoS_2. These models were constructed using supercells containing two layers each of MoS_2 and K_2S, arranged sequentially, with 132 and 168 atoms in the respective supercells. The Brillouin zone was sampled using 2×3×2 and 2×2×1 Monkhorst-Pack k-point grids for K_2S-intercalated 1T'- and 2H-MoS_2, respectively. Experimental results reveal that the interlayer distance of synthesized K_2S-intercalated MoS_2 extends from 5.88 Å (intrinsic 1T'-MoS_2) to 9.06 Å, an increase of about 3.2 Å. In comparison, K-intercalated MoS_2 exhibits a similar interlayer distance increase of about 2 Å.[5] Given that the K-K distance in the K_2S cubic bulk is approximately 3.7 Å, we hypothesize that the K_2S intercalation layer forms an approximate monolayer structure between MoS_2 layers, similar to the K intercalation layer in K-intercalated MoS_2. Previous theoretical studies have predicted K_2S to be stable as a 1T monolayer structure.[6] Considering this, models of K_2S-intercalated 1T'-MoS_2 and K_2S-intercalated 2H-MoS_2 were constructed, denoted as 1T'-$(K_2S)_{x=0.22}MoS_2$ and 2H-$(K_2S)_{x=0.17}MoS_2$, respectively, with the chemical percentage of K_2S

approximating the experimental observation (x=0.18). These models are shown in Figure S12. In these models, K_2S formed a 1T monolayer intercalated between two layers of MoS_2, creating a sandwich-like structure. Upon structural relaxation, the K_2S intercalation layer relaxed to a nearly flat monolayer in 1T'-$(K_2S)_{x=0.22}MoS_2$, with the interlayer distance of MoS_2 reaching 8.10 Å, closely matching the experimental value of 9.06 Å. In contrast, the 2H-$(K_2S)_{x=0.17}MoS_2$ model exhibited an interlayer distance of 11.4 Å, deviating from the experimental observation. The average bond lengths of Mo-Mo and Mo-S in MoS_2 after K_2S intercalation were also determined, as shown in Table S1. The results indicate that K_2S intercalation has minimal impact on the in-plane structure of both 2H and 1T' MoS_2, as the average bond lengths of Mo-Mo and Mo-S remain nearly unchanged. This is consistent with experimental findings, where TEM and XRD data suggest that the in-plane structure of 1T' MoS_2 is preserved after intercalation.

Table S1. Average bond length of Mo-Mo and Mo-S of MoS_2 before and after K_2S intercalation acquired by theoretical calculation.

**average bond length (Å)**	**2H MoS_2**	**K_2S-intercalated 2H MoS_2**	**1T' MoS_2**	**K_2S-intercalated 1T' MoS_2**
Mo-Mo	3.16	3.17	2.79	2.81
Mo-S	2.40	2.41	2.43	2.44

The formation energy of K_2S-intercalated MoS_2 systems were then calculated using the expression:

$$\boldsymbol{E}_{f(x)} = \boldsymbol{E}_{\mathbf{X-(K_2S)}_x\mathbf{MoS_2}} - \boldsymbol{E}_{\mathbf{X-MoS_2}} - x\boldsymbol{E}_{\mathbf{K_2S}}$$

where $\boldsymbol{E}_{\mathbf{X-(K_2S)}_x\mathbf{MoS_2}}$ represents the total energy of the K_2S-intercalated MoS_2 system in phase X (1T' or 2H), $\boldsymbol{E}_{\mathbf{X-MoS_2}}$ is the total energy of MoS_2 in the same

phase, and $\boldsymbol{E_{K_2S}}$ is the total energy of the K_2S cubic bulk. The formation energies of K_2S-intercalated 1T'- and 2H-MoS_2 models were -0.077 eV/atom and 0.076 eV/atom, respectively, indicating that the K_2S-intercalated 1T'-MoS_2 is more energetically stable.

This increased stability of 1T' phase over the 2H phase is attributed primarily to the interlayer confinement effect of K_2S intercalation and enhanced N-doping effect in the MoS_2. To investigate the interlayer confinement effect, we have analyzed the average bond lengths of K-S within K_2S layer (intralayer) and between K_2S and MoS_2 layers (interlayer). For 1T' phase, the average intralayer K-S bond length was about 3.42 Å, and interlayer K-S bond length was about 3.26 Å. For 2H phase, the intralayer K-S bond length averaged to about 3.08 Å, while the interlayer K-S bond length was about 3.34 Å. The shorter interlayer K-S bond length in the 1T' phase implies stronger interlayer interactions, enhancing the confinement effect of K_2S intercalation within the 1T'-MoS_2 structure. To investigate charge transfer in the K_2S-intercalated MoS_2, we have conducted the bader charge analysis and summarized the results in Table S2 and Table S3. The analysis revealed that charge transfer occurs primarily from the S atoms of K_2S layer to the S atoms in the MoS_2 layer, with a significantly larger magnitude for the 1T' phase (-1.04 electrons per S atom) compared to the 2H phase (-0.46 electrons per S atom). Charge density difference plots further confirmed enhanced N-doping in the 1T'-MoS_2, as show in Figure S13. To investigate the influence of charge transfer on structural stability, we have performed electron doping calculations on pristine 1T'- and 2H-MoS_2, as shown in Figure S14. The results indicated that increasing electron doping concentration favored the stability of the 1T' phase over the 2H phase, affirming that enhanced N-doping effect in the K_2S-intercalated 1T'- MoS_2 contributes significantly to its stability.

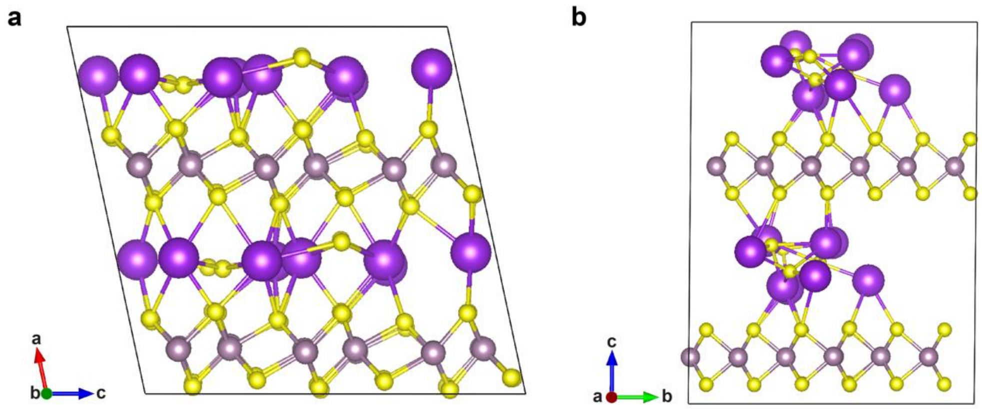

Figure S12. The K_2S-intercalated MoS_2 models. **(a)** 1T'-$(K_2S)_{x=0.22}MoS_2$ and **(b)** 2H-$(K_2S)_{x=0.17}MoS_2$ models.

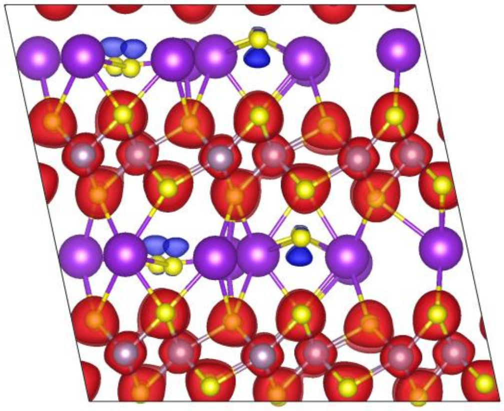

Figure S13. Isosurface plots of charge density differences for the 1T'-$(K_2S)_{x=0.22}MoS_2$ models. The isosurface densities for the charge accumulation region (red color) and depletion region (bule color) are 0.15 e/Bohr^3 and 0.01 e/Bohr^3, respectively.

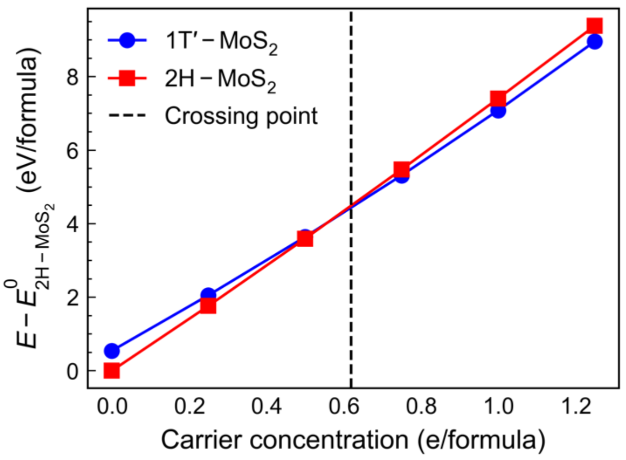

Figure S14. Energy difference between the pristine 1T'- and 2H-MoS_2 models as a function of increasing electron doping concentration.

Table S2. Bader charge analysis of the K_2S-intercalated 1T' MoS_2 model.

bader charge (e)	Mo	S	K	S
K_2S-intercalated 1T' MoS_2	4.327	6.967	8.179	6.467
K_2S (cubic-bulk)			8.246	7.508
MoS_2 (1T'-bulk)	4.324	6.838		
delta_e	0.003	0.129	-0.067	-1.041

Table S3. Bader charge analysis of the K_2S-intercalated 2H MoS_2 model.

bader charge (e)	Mo	S	K	S
K_2S-intercalated 2H MoS_2	4.255	6.919	8.195	7.052

K_2S (cubic-bulk)			8.246	7.508
MoS_2 (2H-bulk)	4.308	6.846		
delta_e	-0.053	0.073	-0.051	-0.456

A piece of K_2S intercalated 1T' MoS_2 flake was transferred on gold electrodes which were fabricated on SiO_2/Si substrate by conventional photolithography process. The Figure S15a shows its optical image. The output characteristic curve and transfer characteristic curve were measured by a semiconductor analyzer (Keysight B1500A) with a probe station (LakeShore). As shown in Figure S15b, the linear output characteristic curve suggests there is Ohmic contact between gold electrodes and K_2S intercalated 1T' MoS_2. The transfer characteristic curve (Figure S15c) shows no gate-controlled drain current, which also confirms the K_2S intercalated 1T' MoS_2 is metallic.

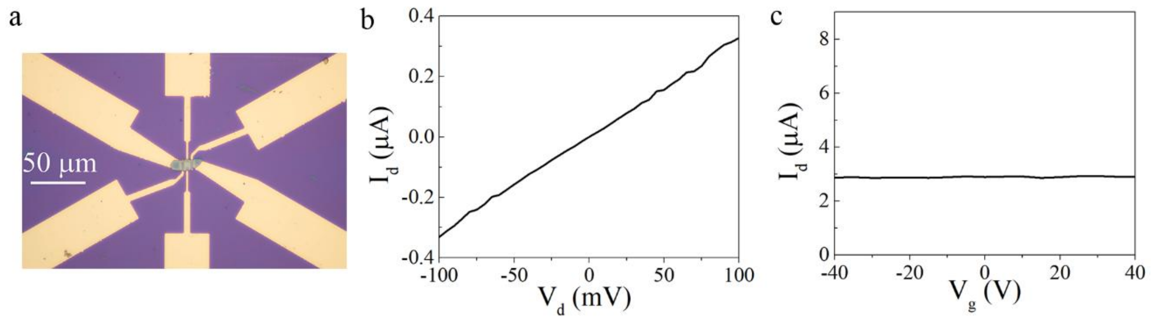

Figure S15. a, Optical image of K_2S intercalated 1T' MoS_2 transferred on gold electrodes. b, Output characteristic curve of K_2S intercalated 1T' MoS_2. c, Transfer characteristic curve of K_2S intercalated 1T' MoS_2.

The Na_2S-intercalated 1T' MoS_2 shows the same Raman spectrum as K_2S-intercalated 1T' MoS_2, as shown in Figure S16, confirming the 1T' phase purity. The Li_2S-intercalated 1T' MoS_2 also has a similar Raman spectrum (Figure S17), but the E_{2g} Raman peak, which is related to 2H MoS_2, appears. The Li is much more active than Na and K, it may react with air when sample is exposed to air. So, part of sample transformed to 2H phase. Nevertheless, the Na_2S-intercalated 1T' MoS_2, like the K_2S-intercalated 1T' MoS_2, possesses excellent crystal quality and phase purity. The TEM and XPS results and discussion are given below.

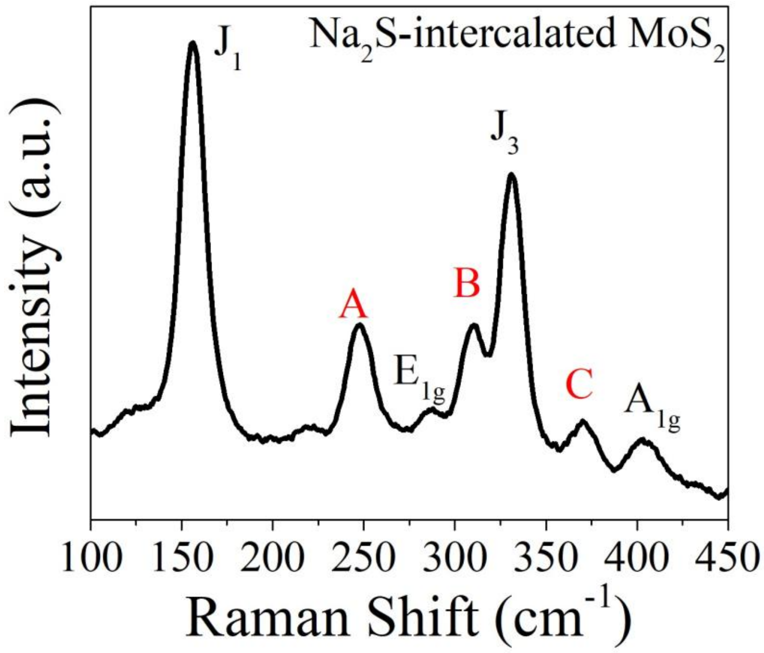

Figure S16. Raman spectrum of Na_2S-intercalated MoS_2.

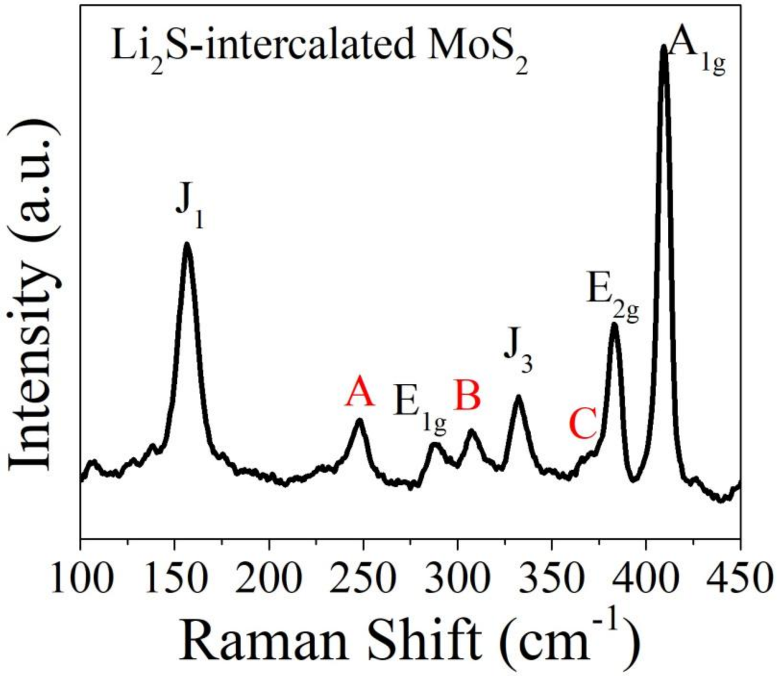

Figure S17. Raman spectrum of Li_2S-intercalated MoS_2.

TEM characterization is conducted to investigate the lattice structure of Na_2S-intercalated 1T' MoS_2, as shown in Figure S18a, b, c. The distinct feature of 1T' phase superstructure, Mo zigzag chains, can be clearly seen in high-resolution TEM image (Figure S18c). The selected area electron diffraction (SAED) pattern confirm the distorted octahedral coordinated structure of 1T' MoS_2 (inset in Figure S18b). As shown in Figure S18d, e, f, g, the EDS mapping result gives evidence of Mo and S element existence, as well as Na element which comes from the intercalation of "Na_2S". These results further confirmed the as-synthesized sample has a 1T' phase MoS_2 structure.

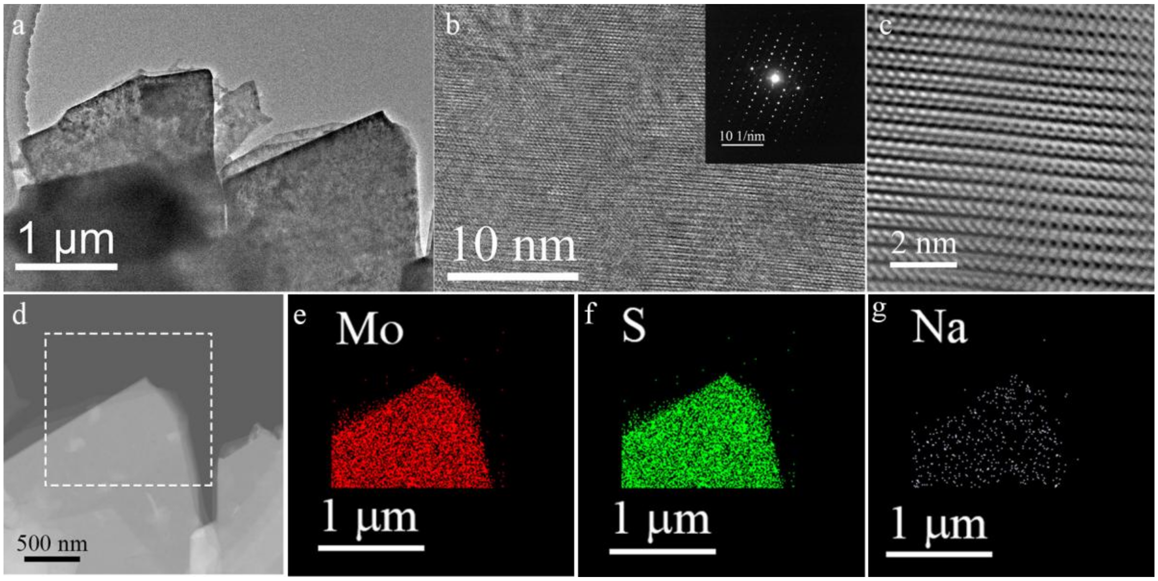

Figure S18. TEM characterization of Na_2S-intercalated 1T' MoS_2. a,b TEM and HRTEM images of Na_2S-intercalated 1T' MoS_2, respectively. Inset in b: SAED pattern of Na_2S-intercalated 1T' MoS_2. c, Fast Fourier transform (FFT) filtered images of b. d, Dark-field STEM image of Na_2S-intercalated 1T' MoS_2. e,f,g, elemental mapping images of Mo, S, and Na, respectively, acquired from rectangular dot box in d.

The Mo 3d XPS spectrum (Figure S19a) shows that both Mo $3d_{5/2}$ and Mo $3d_{3/2}$ peaks have lower binding energy than 2H MoS_2. And the binding energy is nearly the same as the intrinsic 1T' MoS_2[7]. There is only one set of Mo 3d XPS peaks in fitting result, and the fitting curve perfectly fits the experimental curve, which proves the Na_2S-intercalated 1T' MoS_2 is 100 % phase pure. Similar to the K_2S-intercalated 1T' MoS_2, the S 2p XPS spectrum (Figure S19b) shows two sets of peaks, one is corresponding to 1T' MoS_2, the other is corresponding to the intercalated Na_2S. The existence of Na element is also confirmed by Na 1s XPS spectrum (Figure S19c).

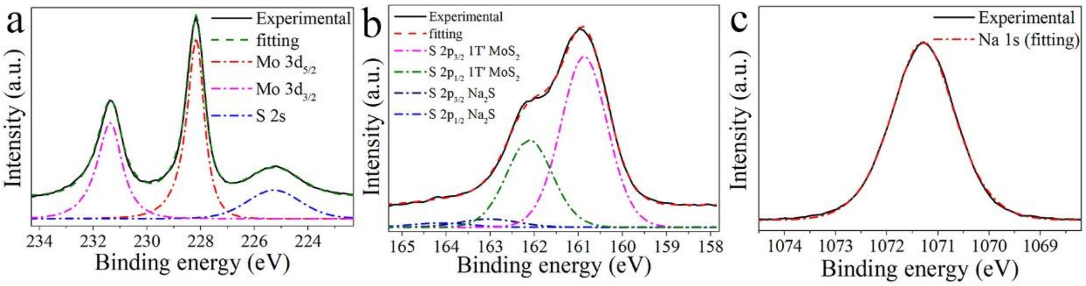

Figure S19. XPS spectra of Na_2S-intercalated 1T' MoS_2. Experimental and fitted XPS (a)Mo 3d and S 2s, (b)S 2p, and (c)Na 1s spectra of Na_2S-intercalated 1T' MoS_2, respectively.

The as-synthesized K_2Se-intercalated $MoSe_2$ Raman spectrum is given in Figure S20. There are J_1, J_2, J_3, and E_{2g}^1 Raman peaks, which are the same as the intrinsic 1T' $MoSe_2$. The additional Raman peaks A and B originated from intercalation. The inset in Figure S20 is the optical image of K_2Se-intercalated $MoSe_2$ flakes on sapphire substrate, showing the parallelogram shape.

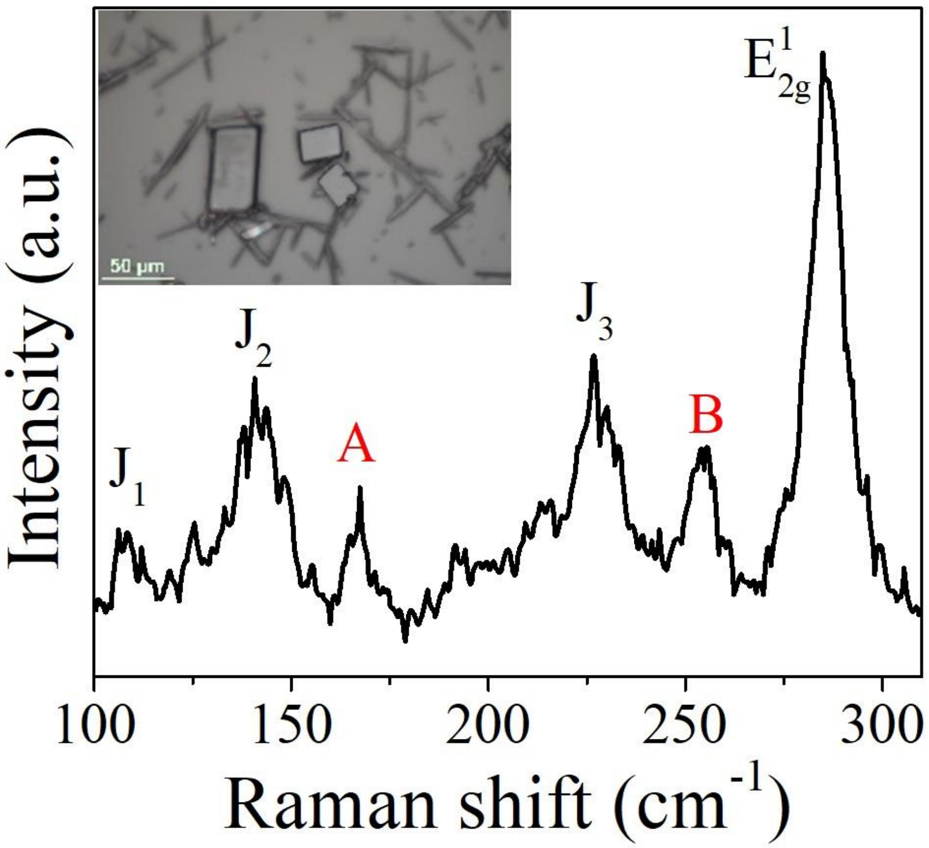

Figure S20. Raman spectrum of K_2Se-intercalated $MoSe_2$. Inset: optical image of K_2Se-intercalated $MoSe_2$.

The Figure S21 shows the Raman spectrum of Na_2Se-intercalated $MoSe_2$, the Raman peak positions are similar to K_2Se-intercalated $MoSe_2$. Similarly, Raman peaks A and B originate from intercalation, while other peaks are close to intrinsic 1T' $MoSe_2$. The optical image of Na_2Se-intercalated $MoSe_2$ flakes is shown in the inset of Figure S21.

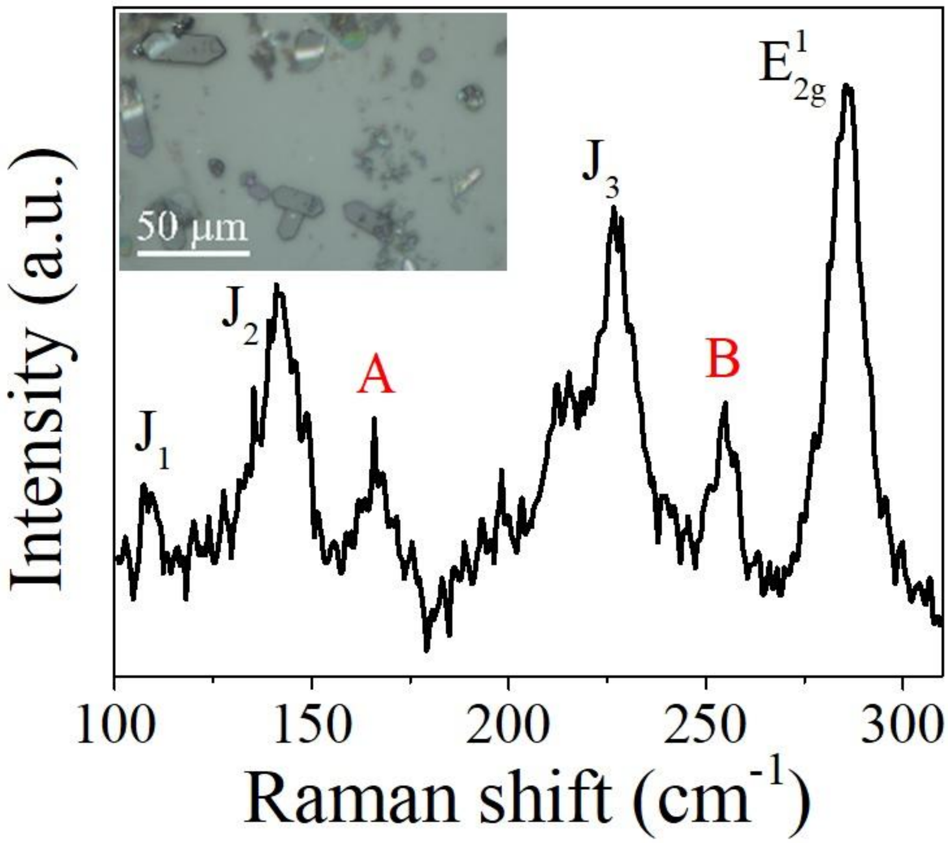

Figure S21. Raman spectrum of Na_2Se-intercalated $MoSe_2$. Inset: optical image of Na_2Se-intercalated $MoSe_2$.

TEM is conducted to characterize the structure of K_2Se-intercalated 1T' $MoSe_2$. The HRTEM image in Figure S22c shows typical Mo zigzag chains of 1T' phase structure. This result proves the flake has 1T' $MoSe_2$ in-plane structure. The element mapping is acquired under dark-field STEM mode, the results are given in Figure S22d, e, f, and g. The Mo, Se, and K element distribution are same as the dark-field STEM image of the flake.

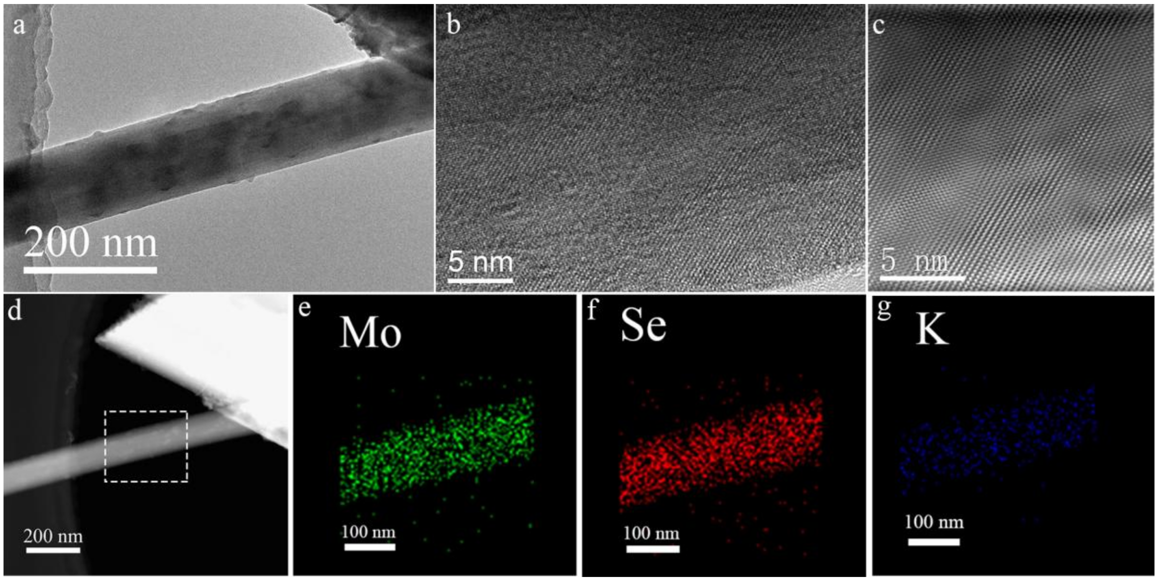

Figure S22. TEM characterization of K_2Se-intercalated 1T’ $MoSe_2$. a,b TEM and HRTEM images of K_2Se-intercalated 1T’ $MoSe_2$, respectively. c, Fast Fourier transform (FFT) filtered images of b. d, Dark-field STEM image of K_2Se-intercalated 1T’ $MoSe_2$. e,f,g, elemental mapping images of Mo, Se, and K, respectively, acquired from rectangular dot box in d.

The as-synthesized K_2Te-intercalated $MoTe_2$ Raman spectrum is given in Figure S23. There are A_g and B_g Raman peaks, the same as the intrinsic 1T’ $MoTe_2$. The additional Raman peaks A originates from intercalation. The inset in Figure S23 is the optical image of K_2Te-intercalated $MoTe_2$ flakes on sapphire substrate.

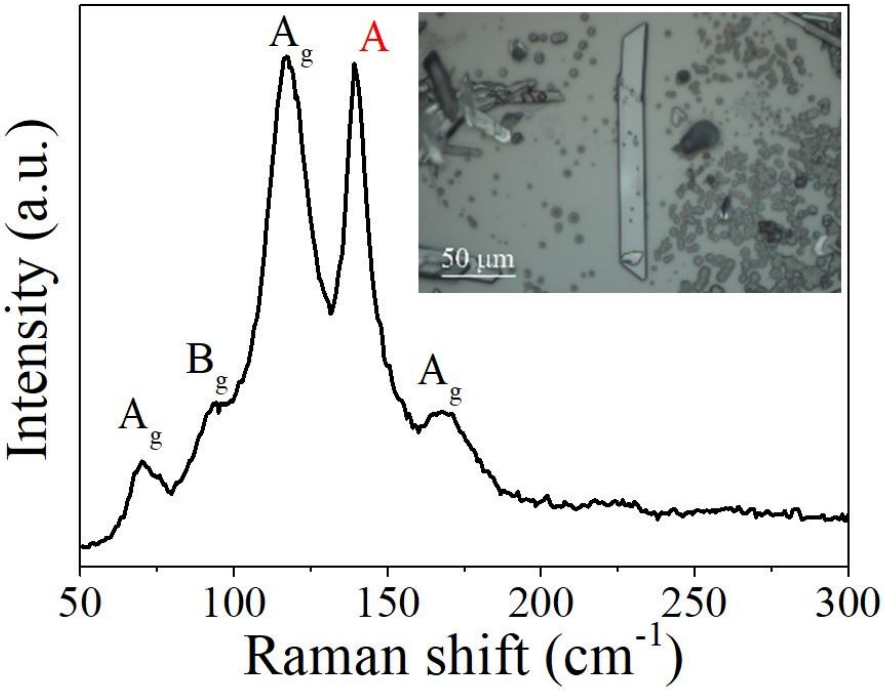

Figure S23. Raman spectrum of K_2Te-intercalated $MoTe_2$. Inset: optical image of K_2Te-intercalated $MoTe_2$.

TEM characterization is conducted to investigate the lattice structure of K_2Te-intercalated 1T' $MoTe_2$, as shown in Figure S24a, b, c. The distinct feature of 1T' phase superstructure can be clearly seen in high-resolution TEM image (Figure S24c). The selected area electron diffraction (SAED) pattern confirms the distorted octahedral coordinated structure of 1T' $MoTe_2$ (inset in Figure S24b). As shown in Figure S24d, e, f, g, the EDS mapping result gives evidence of Mo and Te element existence, as well as K element which comes from the intercalation of "K_2Te". These results confirmed the as-synthesized sample has 1T' phase $MoTe_2$ structure.

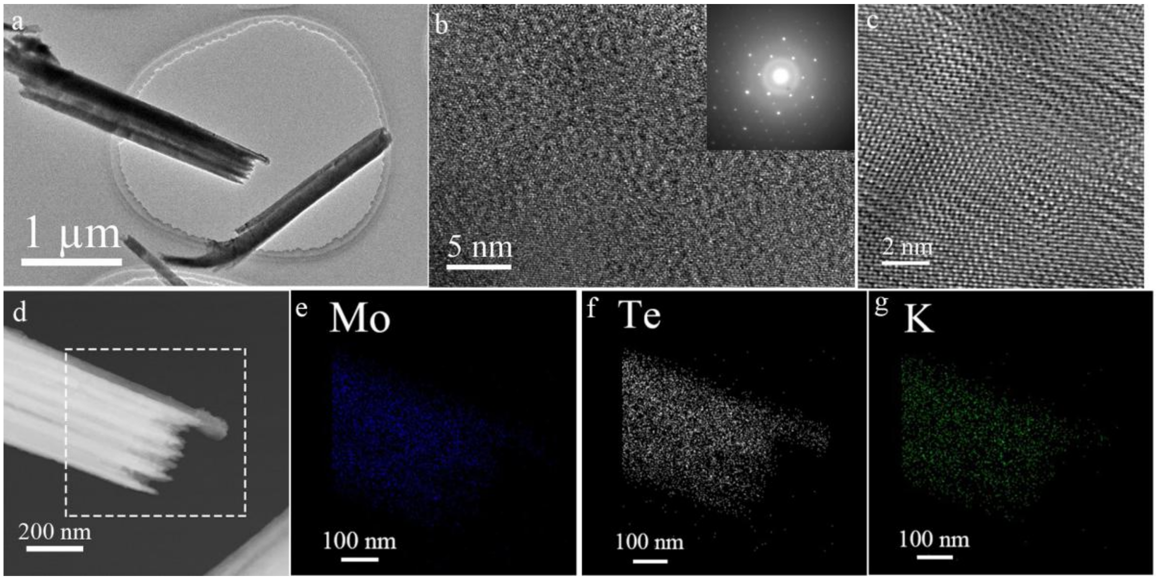

Figure S24. TEM characterization of K_2Te-intercalated 1T' $MoTe_2$. a,b TEM and HRTEM images of K_2Te-intercalated 1T' $MoTe_2$, respectively. Inset in b: SAED pattern of K_2Te-intercalated 1T' $MoTe_2$. c, Fast Fourier transform (FFT) filtered images of b. d, Dark-field STEM image of K_2Te-intercalated 1T' $MoTe_2$. e,f,g, elemental mapping images of Mo, Te, and K, respectively, acquired from rectangular dot box in d.

Supplementary Note 5

In the HER measurement, an electrochemical configuration with H-type cell was employed with 0.5 M H_2SO_4 solution as electrolyte, K_2S-intercalated 1T' MoS_2/carbon cloth as working electrode, a saturated Ag/AgCl reference electrode and a graphite counter electrode. Graphite counter electrode was selected instead of Pt to avoid the Pt deposition on working electrode.[8] The graphite counter electrode was placed in one cell, which was separated from the other cell by a Nafion film. The working electrode and reference electrode were placed together in the other cell. A double bridge structure with Vycor glass junctions was used to set the reference electrode to avoid contamination.[9]

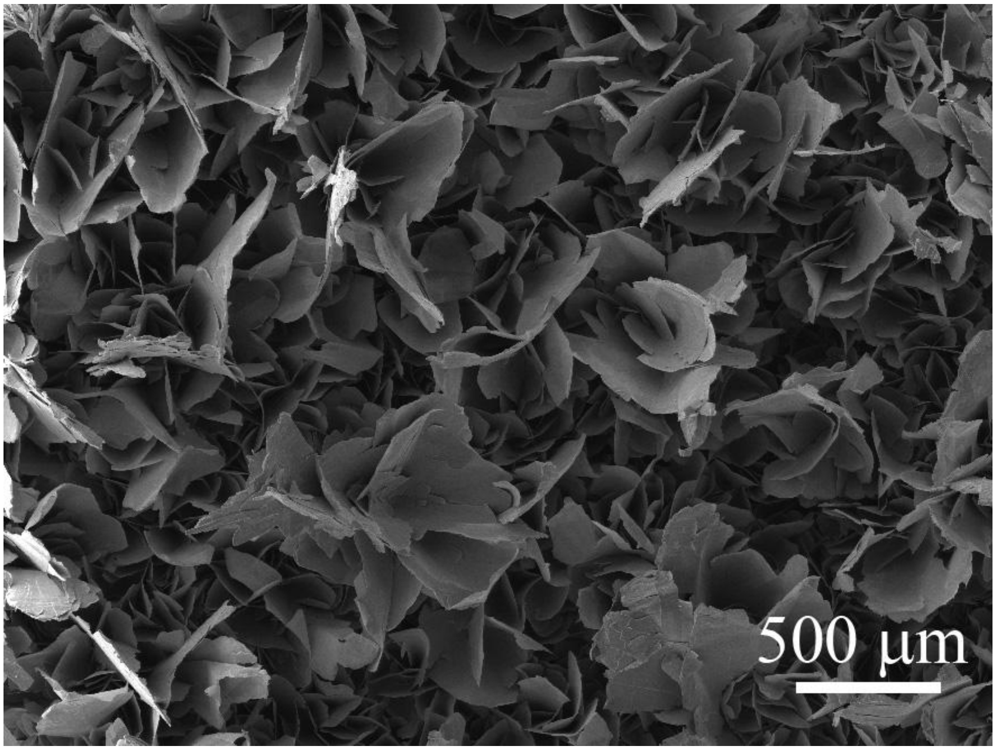

Figure S25. SEM image of K_2S-intercalated 1T' MoS_2/carbon cloth after 30000 cycles, used as HER electrocatalyst.

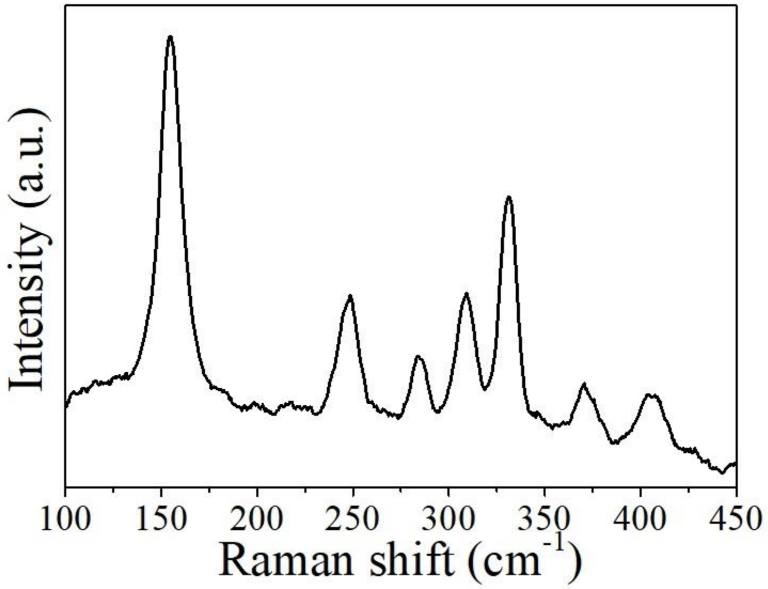

Figure S26. Raman spectrum of K_2S-intercalated 1T' MoS_2/carbon cloth after 30000 cycles, used as HER electrocatalyst.

Table S4. The best TMDs-based HER electrocatalysts via phase engineering.

materials	preparation method	phase purity (%)	electrode	Tafel slope (mV/dec)	overpotential η (mV) at 10 mA/cm^2	stability	Ref.
1T MoS_2 nanodots	n-butyllithium treatment	70	glassy carbon	53	173	no data	[10]
1T MoSSe nanodots	n-butyllithium treatment	67	glassy carbon	40	140	10000 cycles	
1T MoS_2	Li electrochemical intercalation	53.7	mirror polished glassy carbon	44	175	1000 cycles; 20 mA/cm^2 for 5 h	[11]
1T WS_2	n-butyllithium treatment	76	glass carbon	55	Ca. 240	10000 cycles; η = -0.3 V for 120 h	[12]
1T' MoS_2	Lithium intercalation using lithium borohydrate	80	glassy carbon	40	Ca. 200	no data	[13]
1T/2H MoS_2 flake	n-butyllithium treatment	70	Au pad	50	Ca. 210	no data	[14]
1T' WSe_2 nanoflowers	colloidal phase synthesis	not specified	carbon paper	150	300	no data	[15]
1T' MoS_2 flakes	reduction of K_2MoS_4 in H_2	90	highly oriented pyrolytic graphite	51	205	1000 cycles	[16]
1T MoS_2 quantum dots	electrochemical Li intercalation	92−97	carbon fiber paper	44	92	10000 cycles; 200 mA/cm^2 for 80 h	[17]
2M WSe_2 nanosheets	colloidal reaction	not specified	glassy carbon	71	104	10 mA/cm^2 for 120 h	[18]
1T MoS_2 with single-layer hollow structure	Bi ions absorption	not specified	glassy carbon	40	137	30 mA/cm^2 for 36 h	[19]
1T $MoSe_2$–VSe_2–$NbSe_2$ ternary alloy nanosheets	hot-injection colloidal reaction	60	glassy carbon	55	80	20 mA/cm^2 for 120 h	[20]
1T MoS_2 nanoflowers	hydrothermal synthesis	80.5	carbon paper	44	165	1000 cycles	[21]

1T WS_2 nanohelices	aqueous electrochemical activation	70	glassy carbon	40	170	20000 cycles	[22]
1T P-doped WS_2 nanosphere	solvothermal method combined with chemical vapor deposition	82.88	carbon fiber paper	73.73	125	190 mV for 10 h; 1000 cycles	[23]
K_2S-intercalated 1T' MoS_2	K_2MoO_4, S, and H_2 reaction with restricted reaction temperature at 750 °C	100	carbon cloth	39.3	73	30000 cycles; 50 mA/cm^2 for 1000 hours	This work

Table S5. Representative non-precious HER electrocatalysts.

materials	electrode	Tafel slope (mV/dec)	overpotential η (mV) at 10 mA/cm^2	stability	Ref.
CoP nanowire arrays	carbon cloth	51	67	5000 cycles	[24]
FeP nanowire array	Ti plate	38	57	90 mA/cm^2 for 15 h	[25]
$Fe_{0.5}Co_{0.5}P$ nanowire	carbon cloth	30	37	100 mA/cm^2 for 100 h	[26]
se-MoS_2 sheet arrays	carbon paper	56	104	10 mA/cm^2 for 24 h	[27]
perpendicularly oriented $MoSe_2$/graphene nanosheets	graphite	61	159	150 mV for 100 min	[28]
CoS_2 micro- and nanostructures	graphite	51.6	145	10 mA/cm^2 for 40 h	[29]
molybdenum phosphosulfide film	Ti foil	50	64	1000 cycles	[30]
$Ni_{0.9}Fe_{0.1}PS_3$ nanosheets	glassy carbon	73	72	no data	[31]
cobalt encapsulated by N, B codoped ultrathin carbon cages	glassy carbon	63.7	96	200 mV for 10 h	[32]
NiFe layered double hydroxide nanosheets	nickel foam	62.3	59	1.7 V for 100 h	[33]
Mott-Schottky heterojunction of semi-conductive MoS_2 nanoparticles/metallic CoS_2 nanotubes	carbon cloth	64.4	99.3	185.8 mV for 50 h	[34]
carbon-supported MoS_x nanocomposites	glassy carbon	50.03	184	1000 cycles	[35]
Fe/Mg-N_4/nitrogen-doped porous carbon	glassy carbon	47.9	21	10 mA/cm^2 for 80 h	[36]
iron-incorporated molybdenum oxide	Ni foam	71	17	200 mA/cm^2 for 200 h	[37]
cobalt phosphide heterojunctions	carbon paper	106.54	97	50 mA/cm^2 for 24 h	[38]

NiMo/Ni_2P heterojunction	Ni foam	29.45	15	5000 cycles; 500 mA/cm^2 for 20 h	[39]
K_2S-intercalated 1T' MoS_2	carbon cloth	39.3	73	30000 cycles; 50 mA/cm^2 for 1000 hours	This work

An additional selected area electron diffraction (SAED) pattern of K_2S-intercalated 1T' MoS_2 prepared by this work is shown in Figure S27. There is only one set of SAED pattern. It shows the distorted octahedral coordinated structure of 1T' MoS_2, which is different from the hexagonal pattern of 2H phase. This result confirms, at less in the tested area, the sample is 1T' MoS_2 single crystal. If the sample is the mixture of 1T' and 2H phase, two sets of patterns overlay. This result could be one of the evidences to support the sample is very close to 100 % 1T' phase purity.

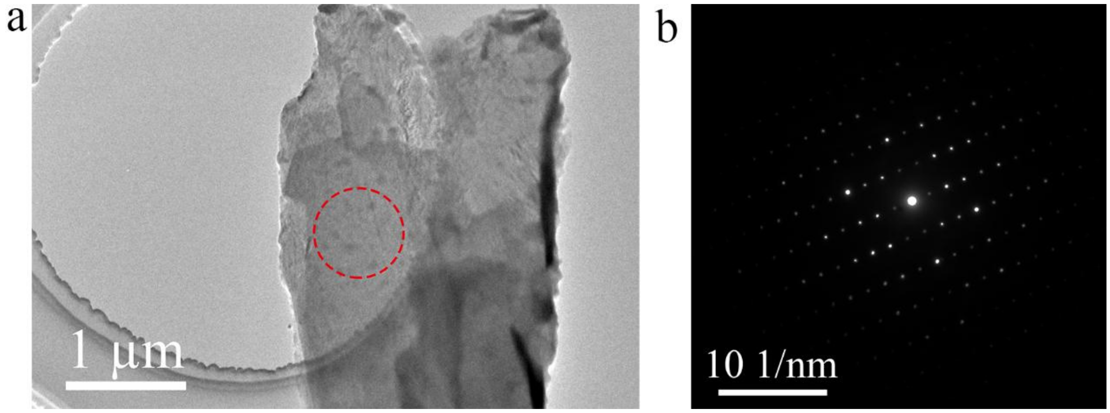

Figure S27. An additional selected area electron diffraction (SAED) test result of K_2S-intercalated 1T' MoS_2. a) TEM image of a piece of K_2S-intercalated 1T' MoS_2 flake. b) SAED pattern acquired from red circle area shown in a.

References

[1] a)G. Kresse, D. Joubert, *Phys. Rev. B* **1999**, 59, 1758; b)G. Kresse, J. Furthmuller, *Phys. Rev. B* **1996**, 54, 11169.

[2] J. P. Perdew, K. Burke, M. Ernzerhof, *Phys. Rev. Lett.* **1996**, 77, 3865.

[3] S. Grimme, J. Antony, S. Ehrlich, H. Krieg, *J. Chem. Phys.* **2010**, 132, 19, 154104.

[4] a)C. D. West, *Z. Für Krist. - Cryst. Mater.* **1934**, 88, 97; b)Z. C. Lai, Q. Y. He, T. Ha Tran, D. V. M. Repaka, D. D. Zhou, Y. Sun, S. B. Xi, Y. X. Li, A. Chaturvedi, C. L. Tan, B. Chen, G. H. Nam, B. Li, C. Y. Ling, W. Zhai, Z. Y. Shi, D. Y. Hu, V. Sharma, Z. N. Hu, Y. Chen, Z. C. Zhang, Y. F. Yu, X. R. Wang, R. V. Ramanujan, Y. M. Ma, K. Hippalgaonkar, H. Zhang, *Nat. Mater.* **2021**, 20, 1113.

[5] R. Y. Zhang, I. L. Tsai, J. Chapman, E. Khestanova, J. Waters, I. V. Grigorieva, *Nano Lett.* **2016**, 16, 629.

[6] J. Zeng, M. Lu, H. W. Liu, H. Jiang, X. C. Xie, *Sci. Bull.* **2021**, 66, 765.

[7] L. N. Liu, J. X. Wu, L. Y. Wu, M. Ye, X. Z. Liu, Q. Wang, S. Y. Hou, P. F. Lu, L. F. Sun, J. Y. Zheng, L. Xing, L. Gu, X. W. Jiang, L. M. Xie, L. Y. Jiao, *Nat. Mater.* **2018**, 17, 1108.

[8] G. F. Dong, M. Fang, H. T. Wang, S. Yip, H. Y. Cheung, F. Y. Wang, C. Y. Wong, S. T. Chu, J. C. Ho, *J. Mater. Chem. A* **2015**, 3, 13080.

[9] S. G. Ji, H. Kim, C. Park, W. Kim, C. H. Choi, *ACS Catal.* **2020**, 10, 10773.

[10] C. L. Tan, Z. M. Luo, A. Chaturvedi, Y. Q. Cai, Y. H. Du, Y. Gong, Y. Huang, Z. C. Lai, X. Zhang, L. R. Zheng, X. Y. Qi, M. H. Goh, J. Wang, S. K. Han, X. J. Wu, L. Gu, C. Kloc, H. Zhang, *Adv. Mater.* **2018**, 30, 9, 1705509.

[11] H. T. Wang, Z. Y. Lu, S. C. Xu, D. S. Kong, J. J. Cha, G. Y. Zheng, P. C. Hsu, K. Yan, D. Bradshaw, F. B. Prinz, Y. Cui, *Proc. Natl. Acad. Sci. U. S. A.* **2013**, 110, 19701.

[12] D. Voiry, H. Yamaguchi, J. W. Li, R. Silva, D. C. B. Alves, T. Fujita, M. W. Chen, T. Asefa, V. B. Shenoy, G. Eda, M. Chhowalla, *Nat. Mater.* **2013**, 12, 850.

[13] D. Voiry, M. Salehi, R. Silva, T. Fujita, M. W. Chen, T. Asefa, V. B. Shenoy, G. Eda, M. Chhowalla, *Nano Lett.* **2013**, 13, 6222.

[14] D. Voiry, R. Fullon, J. E. Yang, C. Silva, R. Kappera, I. Bozkurt, D. Kaplan, M. J. Lagos, P. E. Batson, G. Gupta, A. D. Mohite, L. Dong, D. Q. Er, V. B. Shenoy, T. Asefa, M. Chhowalla, *Nat. Mater.* **2016**, 15, 1003.

[15] M. S. Sokolikova, P. C. Sherrell, P. Palczynski, V. L. Bemmer, C. Mattevi, *Nat. Commun.* **2019**, 10, 8, 712.

[16] L. N. Liu, J. X. Wu, L. Y. Wu, M. Ye, X. Z. Liu, Q. Wang, S. Y. Hou, P. F. Lu, L. F. Sun, J. Y. Zheng, L. Xing, L. Gu, X. W. Jiang, L. M. Xie, L. Y. Jiao, *Nat. Mater.* **2018**, 17, 1108.

[17] W. S. Chen, J. J. Gu, Q. L. Liu, R. C. Luo, L. L. Yao, B. Y. Sun, W. Zhang, H. L. Su, B. Chen, P. Liu, D. Zhang, *ACS Nano* **2018**, 12, 308.

[18] I. S. Kwon, I. H. Kwak, J. Y. Kim, S. J. Lee, Q. A. Sial, J. Ihsan, K. S. Lee, S. J. Yoo, J. Park, H. S. Kang, *Adv. Mater.* **2023**, 11, 2307867.

[19] B. J. Li, K. K. Nie, Y. J. Zhang, L. X. Yi, Y. L. Yuan, S. K. Chong, Z. Q. Liu, W. Huang, *Adv. Mater.* **2023**, 10.

[20] I. S. Kwon, I. H. Kwak, G. M. Zewdie, S. J. Lee, J. Y. Kim, S. J. Yoo, J. G. Kim, J. Park, H. S. Kang, *Adv. Mater.* **2022**, 34, 9, 2205524.

[21] X. T. Li, X. N. Sun, H. F. Yu, H. T. Li, X. Y. Sun, X. Tao, Y. Z. Zheng, *Appl. Catal. B-Environ.* **2022**, 307, 12, 121156.

[22] L. L. Wang, G. Zhou, H. Luo, Q. F. Zhang, J. Wang, C. W. Zhao, A. M. Rao, B. Xu, B. A. Lu, *Appl. Catal. B-Environ.* **2019**, 256, 7, 117802.

[23] L. Sun, M. Y. Gao, Z. X. Jing, Z. Y. Cheng, D. H. Zheng, H. Z. Xu, Q. N. Zhou, J. J. Lin, *Chem. Eng. J.* **2022**, 429, 9, 132187.

[24] J. Q. Tian, Q. Liu, A. M. Asiri, X. P. Sun, *J. Am. Chem. Soc.* **2014**, 136, 7587.

[25] P. Jiang, Q. Liu, Y. H. Liang, J. Q. Tian, A. M. Asiri, X. P. Sun, *Angew. Chem.-Int. Edit.* **2014**, 53, 12855.

[26] C. Tang, L. F. Gan, R. Zhang, W. B. Lu, X. E. Jiang, A. M. Asiri, X. P. Sun, J. Wang, L. Chen, *Nano Lett.* **2016**, 16, 6617.

[27] J. Hu, B. L. Huang, C. X. Zhang, Z. L. Wang, Y. M. An, D. Zhou, H. Lin, M. K. H. Leung, S. H. Yang, *Energy Environ. Sci.* **2017**, 10, 593.

[28] S. Mao, Z. H. Wen, S. Q. Ci, X. R. Guo, K. Ostrikov, J. H. Chen, *Small* **2015**, 11, 414.

[29] M. S. Faber, R. Dziedzic, M. A. Lukowski, N. S. Kaiser, Q. Ding, S. Jin, *J. Am. Chem. Soc.* **2014**, 136, 10053.

[30] J. Kibsgaard, T. F. Jaramillo, *Angew. Chem.-Int. Edit.* **2014**, 53, 14433.

[31] B. Song, K. Li, Y. Yin, T. Wu, L. N. Dang, M. Cabán-Acevedo, J. C. Han, T. L. Gao, X. J. Wang, Z. H. Zhang, J. R. Schmidt, P. Xu, S. Jin, *ACS Catal.* **2017**, 7, 8549.

[32] H. B. Zhang, Z. J. Ma, J. J. Duan, H. M. Liu, G. G. Liu, T. Wang, K. Chang, M. Li, L. Shi, X. G. Meng, K. C. Wu, J. H. Ye, *ACS Nano* **2016**, 10, 684.

[33] Z. Qiu, C. W. Tai, G. A. Niklasson, T. Edvinsson, *Energy Environ. Sci.* **2019**, 12, 572.

[34] T. L. L. Doan, D. C. Nguyen, K. Kang, A. Ponnusamy, H. I. Eya, N. Y. Dzade, C. S. Kim, C. H. Park, *Appl. Catal. B-Environ. Energy* **2024**, 342, 17, 123295.

[35] Q. M. Liu, F. Nichols, A. Bhuller, K. Singewald, H. L. Kuo, J. Q. Lu, G. L. Millhauser, F. Bridges, Q. F. Ge, S. W. Chen, *Appl. Catal. B-Environ. Energy* **2024**, 342, 11, 123399.

[36] R. Nivetha, M. R. Asrami, R. Kumar, S. Sharma, M. Jourshabani, R. D. Kumar, S. Ravichandran, B. K. Lee, Y. Lee, J. S. Chung, S. G. Kang, W. M. Choi, S. H. Hur, *Small Struct.* **2023**, 14.

[37] J. P. Sun, S. Y. Qin, Z. Zhao, Z. S. Zhang, X. C. Meng, *Mater. Horizons* **2023**, 13.

[38] X. F. Cao, J. Z. Tian, Y. Tan, Y. C. Zhu, J. Hu, Y. Wang, E. Z. Liu, Z. Chen, *Small* **2023**, 9.

[39] Y. F. Yang, X. Y. Li, G. L. Liu, H. X. Liu, Y. H. Shi, C. M. Ye, Z. Fang, M. X. Ye, J. F. Shen, *Adv. Mater.* **2023**, 12.
